# Simultaneous tissue profiling of eicosanoid and endocannabinoid lipid families in a rat model of osteoarthritis[S]

**DOI:** 10.1194/jlr.M048694

**Published:** 2014-09

**Authors:** Amy Wong, Devi R. Sagar, Catharine A. Ortori, David A. Kendall, Victoria Chapman, David A. Barrett

**Affiliations:** * Centre for Analytical Bioscience, School of Pharmacy, University of Nottingham, Nottingham NG7 2UH, UK; † School of Life Sciences, University of Nottingham, Nottingham NG7 2UH, UK; § Arthritis Research UK Pain Centre, University of Nottingham, Nottingham NG7 2UH, UK

**Keywords:** mass spectrometry, lipidomics, metabolic profiling, brain regions, knee, blood plasma

## Abstract

We describe a novel LC method for the simultaneous and quantitative profiling of 43 oxylipins including eicosanoids, endocannabinoids, and structurally related bioactive lipids with modified acyl groups. The LC-MS/MS method uses switching at a defined time between negative and positive electrospray ionization modes to achieve optimal detection sensitivity for all the lipids. The validated method is linear over a range of 0.01–5 nmol/g (0.1–50 nmol/g for 2-arachidonoyl glycerol) with intra- and interday precision and accuracy between 1.38 and 26.76% and 85.22 and 114.3%, respectively. The method successfully quantified bioactive lipids in different tissue types in the rat, including spinal cord, dorsal root ganglia (DRGs), knee joint, brain, and plasma. Distinct regional differences in the pattern of lipid measured between tissue types were observed using principle component analysis. The method was applied to analyze tissue samples from an established preclinical rat model of osteoarthritis (OA) pain and showed that levels of 12-hydroxyeicosatetraenoic acid were significantly increased in the OA rat knee joint compared with controls, and that 15-hydroxyeicosatetraenoic acid was significantly increased in the DRGs in the model of OA compared with controls. The developed LC-MS/MS method has the potential to provide detailed pathway profiling in tissues and biofluids where the disruption of bioactive oxylipins may be involved in disease states.

Biological processes such as inflammation and pain, which involve lipid mediators, are complex events and often include significant regional and/or temporal changes in the levels of many lipid species. Such lipid-mediated cellular processes also underpin normal cell (and thus tissue) homeostasis as well as tissue development, repair, and immunity. Direct or indirect interference of these lipid signaling processes can severely disrupt cellular signaling processes, potentially leading to a range of developmental, autoimmune, cancer, and inflammatory diseases. Two major lipid signaling systems, the eicosanoids and the endocannabinoids, form elements of the oxylipin family and are based primarily on metabolic derivatives of arachidonic acid (AA) and show significant interrelation in terms of enzyme pathways [cyclooxygenase (COX), lipoxygenase (LOX), cytochrome P450 (CYP), and prostaglandin (PG) synthase series of enzymes] involved in the generation of bioactive species (**Fig. 1**). PGs, leukotrienes (LTs), thromboxanes (TXs), HETEs, epoxyeicosatrienoic acids (EETs), hydroperoxyeicosatetraenoic acids (HPETEs), and the equivalent acyl chain modified species including the endocannabinoids, anandamide (AEA), and 2-arachidonoylglycerol (2-AG) are generated from AA by these pathways (Fig. 1). The physiological and biochemical functions of eicosanoid lipids derived from these metabolic routes have been widely studied, but there is significantly less information regarding the equivalent acyl-modified lipids, such as metabolized forms of AEA and 2-AG species and the potential crosstalk/interaction between these two sets of bioactive lipids (1).

**Fig. 1.**
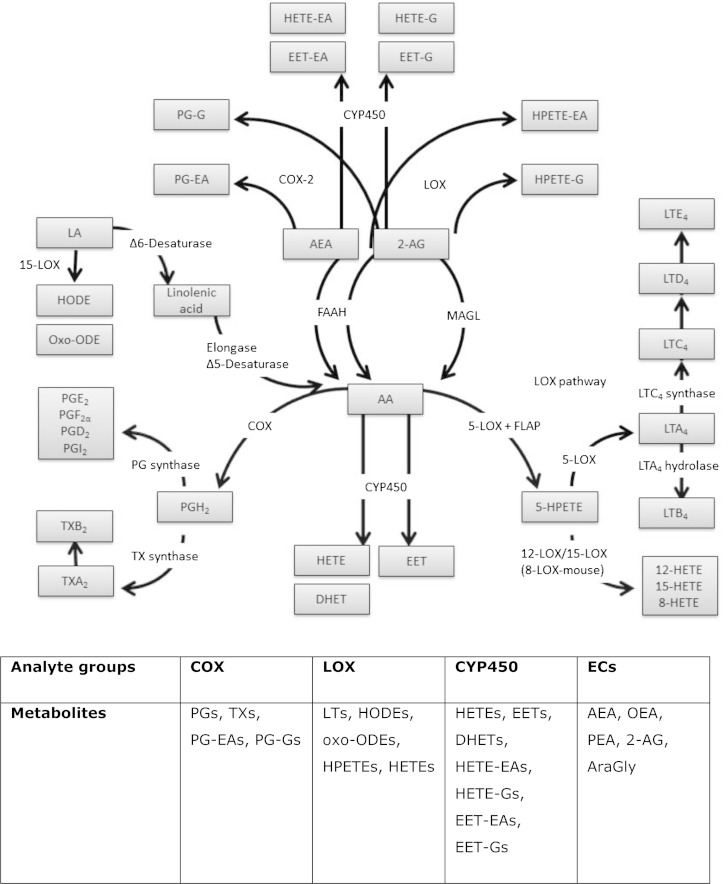
Metabolic pathways of bioactive lipids analyzed with this LC-MS/MS method. G, glycerol; CYP450, cytochrome P450; FAAH, fatty acid amide hydrolase; MAGL, monoacylglycerol lipase; FLAP, 5-LOX activating protein.

The production of pro-inflammatory (e.g., LTs and PGs) and/or anti-inflammatory [e.g., PGs and LXs (2, 3)] eicosanoids, as well as other bioactive lipids, increases during inflammation. Linoleic acid (LA) is a key source of AA and has a direct effect on eicosanoid synthesis (4, 5), as well as being the substrate for the generation of HODEs, which are oxidized to oxooctadecadienoic acids (oxoODEs) by 15-LOX and have anti- and/or pro-inflammatory effects (6). The endocannabinoids and endocannabinoid-like compounds include AEA, 2-AG, pal­mitoyl ethanolamide (PEA), oleoyl ethanolamide (OEA), arachidonoyl glycine (AraGly), and N-arachidonoyl dopamine (NADA). Both the endocannabinoids and eicosanoids are synthesized on demand and are distributed widely in different tissues in the body (7).

Eicosanoids and endocannabinoids mainly target local G protein-coupled receptors such as the prostanoid receptors, cannabinoid receptors, CB_1_ and CB_2_, or nuclear receptors, such as PPARs (8). Several bioactive lipids (12-HPETE, 12-HETE, 9-HODE, 13-HODE, 9-oxoODE, and 13-oxoODE) also activate transient receptor potential vanilloid 1 (TRPV1) (9–13), a nonselective ion channel located on sensory neurons which are activated by thermal, chemical, and painful stimuli (14).

Although LC-MS/MS methods have been developed for the quantification of bioactive lipids, they have typically been limited to a relatively small number of lipids, and/or aimed at a specific type of tissue (15, 16), or multiple methods have been used (17). Successful development of comprehensive quantitative profiling methods for these groups of lipids has been hampered by their relatively low concentrations in tissues (picomolar or nanomolar), structural similarity/isomerization, and incompatibility of MS detection of the different classes of lipids. Methods have been published to quantify the eicosanoids (18–23) but, apart from one method which separated four PGs and their glyceryl esters (24), there are no methods which include the simultaneous analysis of a broad range of eicosanoids and their acyl derivatives such as N-ethylamine or monoacylglycerol derivatives. The main reason for this can be traced to a distinct difference in electrospray ionization MS response of the two core chemical species with those having a free carboxylic acid preferring negative ionization mode and those with modified carboxylic acids preferring positive ionization mode (25–28). The LC-MS/MS method reported here for simultaneous measurement of both of these types of lipids in a single analysis involves staged switching between positive and negative modes of ionization and can be achieved on any standard triple quadrupole LC-MS/MS instrument.

We report a fully validated and quantitative method for the simultaneous measurement of a wide range of bioactive lipids in a typical range of different tissue types in the rat, and demonstrate its application in the detection of changes in peripheral and central tissue oxylipin profiles in a rat model of osteoarthritis (OA).

## MATERIALS AND METHODS

### Chemicals

**Fig. 2.**
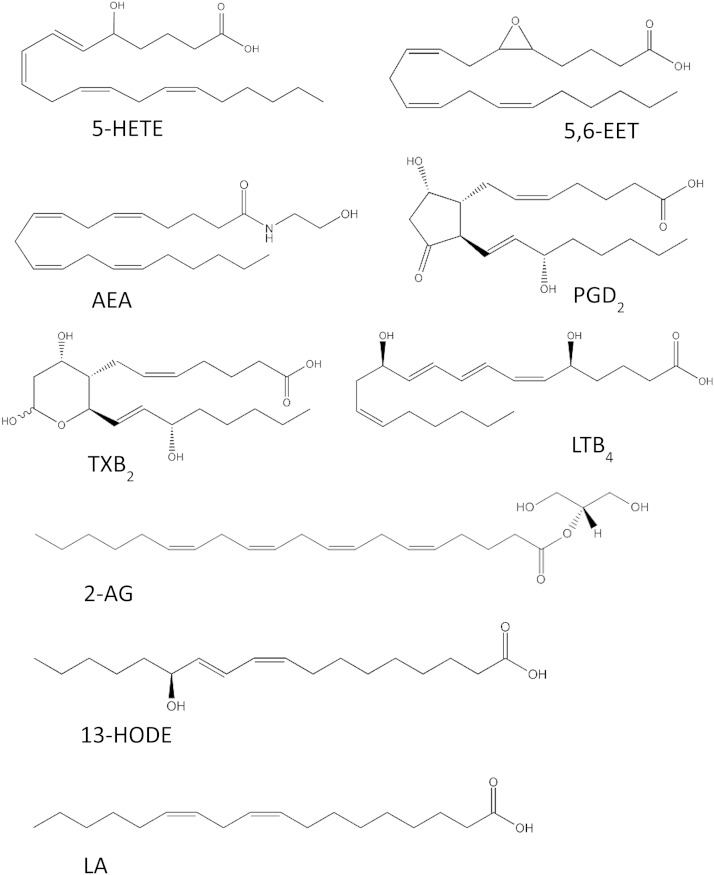
Representative chemical structures of the different groups of lipids measured using LC-MS/MS.

Acetonitrile, ammonium hydroxide, ethanol, ethyl acetate, hexane, formic acid, and methanol were all purchased from Fisher Scientific (Loughborough, UK). All solvents were HPLC grade and far UV grade acetonitrile was also used. Representative chemical structures of the different groups of lipids measured are shown in **Fig. 2**. A full list of the quantified lipids and the sources of standards is provided in the supplementary material.

### Sample preparation

Male Sprague-Dawley rats (200–250 g, Charles River Laboratories) were used and all experiments were conducted in accordance with the UK Home Office regulations. Animals were euthanized by stunning and decapitation followed by rapid dissection of spinal cord; brain divided into frontal cortex, midbrain, hippocampus, rest of cortex, and rest of brain regions; knee joints; and dorsal root ganglia (DRGs); and collection of blood and immediate transfer of tissues into liquid nitrogen. Blood was centrifuged (4°C, 4,000 *g*) and the plasma collected and frozen. All samples were stored immediately at −80°C until extraction and analysis.

Brain, spinal cord, and DRGs were weighed and then homogenized with 1 ml added water with hand-held pestles in a glass tubes. Plasma samples did not have additional water added and paw tissue and knee tissue (crushed) required 1 h slow shaking with the extraction solvent. Internal standards [100 μl of 2-AG-d8 (10 μM) and 15 μl of AEA-d8 (28 nM), 10 μl of PGF2a-ethanolamide (EA)-d4 (2.49 μM), 10 μl of AA-d8 (100 nM), 10 μl of PGD2-d4 (1 μM), and 10 μl of 15-HETE-d8 (7.6 μM)] were added to each sample or blank sample (0.2 ml water), with 10 μl of butylhydroxytoluene. Ethyl acetate:hexane 2.5 ml (9:1, v/v) was added, followed by slow vortex-mixing (10 min) and centrifugation (3,200 *g*, 4°C) for 15 min. The supernatant was transferred to a glass tube, the procedure was repeated, and the supernatants were pooled and evaporated under nitrogen. The samples were then reconstituted in 200 μl of acetonitrile:water (50:50, v/v) and analyzed immediately.

### LC-MS/MS method

An Applied Biosystems MDS SCIEX 4000 Q-Trap hybrid triple-quadrupole-linear ion trap mass spectrometer (Applied Biosystems, Foster City, CA) was used in conjunction with a Shimadzu series 10AD VP LC system (Shimadzu, Columbia, MD). Analytes were separated chromatographically using a complex gradient (detailed in supplementary Table I), mobile phase A (0.05% formic acid in water, pH adjusted with dilute ammonium hydroxide), mobile phase B [methanol:acetonitrile (20:80, v/v)], mobile phase C (acetonitrile), and a Phenomenex Luna C18 (150 × 2.0 mm, 3 μm internal diameter) column maintained at 30°C. The autosampler temperature was maintained at 4°C throughout analysis. Multiple reaction monitoring (MRM) of individual compounds in negative and positive mode using specific precursor and product *m/z* ratios allowed simultaneous measurement of endocannabinoids, eicosanoids, and their metabolites in the same method. At 18.5 min the MS ionization mode was changed from negative to positive electrospray ionization mode, to allow the detection of the endocannabinoids and other oxylipins with a modified carboxylic acid group. Source parameters, declustering potential, collision energy, and collision cell exit potential were optimized by direct infusion or injection to maximize sensitivity (**Table 1**). Quantification was performed using Analyst 1.4.1. Identification of each compound in biological tissue was confirmed by LC retention times of each standard and precursor and product ion *m/z* ratios.

**TABLE 1.** Mass spectrometer values for all compounds where the product ions, declustering potential, collision energy, and collision exit potential were optimized increased sensitivity

Analyte	Retention Time (min)	Q1 Mass	Q3 Mass	DP (V)	CE (V)	CXP (V)
PGD_2_	1.9	351.22	271.21	−40	−25	−20.00
PGE_2_	1.9	351.22	271.21	−50	−25	−15.00
TXB_2_	1.9	369.23	169.09	−50	−25	−10.00
PGE_1_-EA	3.7	396.28	360.20	−55	−15	−10.00
PGF_2α_-EA	3.7	396.28	334.21	−70	−25	−10.00
8,15-Di-HETE	4.1	335.23	127.20	−60	−30	−10.00
PGE_2_-EA	4.3	394.26	358.24	−70	−17	−10.00
PGD_2_-EA	4.4	394.26	203.12	−55	−34	−15.00
14,15-DHET	5.0	337.25	207.15	−75	−25	−3.24
LTB_4_	5.6	335.23	335.23	−60	−15	−15.00
LTE_4_	—	438.23	438.23	−50	−12	−10.00
11,12-DHET	6.0	337.25	167.11	−70	−26	−10.90
19-HETE	6.7	319.24	275.20	−85	−27	−7.23
20-HETE	7.2	319.24	289.22	−67	−30	−6.27
13-HODE	8.3	295.23	195.14	−60	−28	−10.00
16-HETE	8.0	319.24	233.15	−65	−20	−3.91
8,9-DHET	8.3	337.25	127.11	−74	−28	−8.41
9-HODE	9.1	295.23	171.10	−60	−30	−10.00
13-oxoODE	9.7	293.21	113.10	−75	−28	−8.00
15-HETE	9.7	319.24	219.14	−65	−18	−3.78
14,15-EET	11.6	319.24	219.14	−72	−15	−3.50
9-oxoODE	10.1	293.21	185.12	−85	−28	−12.00
5,6-DHET	10.4	337.25	145.06	−71	−26	−10.02
11-HETE	10.8	319.24	167.11	−85	−23	−2.01
11,12-EET	12.8	319.24	167.11	−65	−21	−7.89
12-HETE	11.1	319.24	179.11	−70	−21	−3.00
8-HETE	11.7	319.24	155.07	−65	−20	−9.89
8,9-EET	13.4	319.24	155.08	−72	−17	−9.74
9-HETE	11.9	319.24	123.00	−70	−23	−6.60
12-HPETE	12.1	317.23	153.10	−80	−24	−10.75
5-HETE	13.1	319.24	115.04	−64	−20	−7.00
5-HPETE	13.9	317.23	203.18	−75	−30	−12.00
5,6-EET	13.9	319.24	191.18	−70	−16	−13.60
AraGly	16.3	360.25	74.02	−60	−45	−10.00
LA	17.8	279.23	279.23	−60	−15	−7.00
AA	18.0	303.23	259.00	−80	−20	−15.00
14,15-EET-Glycerol	18. 9	395.27	285.21	80	23	10.02
5,6-EET-EA	19.1	364.28	62.06	90	40	9.00
AEA	20.7	348.28	62.06	25	30	8.56
NADA	21.0	440.31	137.06	25	33	23.98
2-AG	21.1	379.28	287.22	88	22	4.90
PEA	21.6	300.28	62.06	25	30	9.22
OEA	21.9	326.30	62.06	25	32	8.19
AEA-d8*^a^*	20.6	356.33	63.03	80	45	15.00
2-AG-d8*^a^*	21.0	387.33	95.11	70	61	7.08
PGD_2_-d4*^a^*	1.9	355.24	193.15	−45	−29	−10.00
PGF_2α_-EA-d4*^a^*	3.7	400.26	338.24	−70	−23	−10.00
15-HETE-d8*^a^*	9.5	327.29	226.25	−67	−20	−3.97
AA-d8*^a^*	17.8	311.28	267.29	−50	−20	−10.00

a Denotes internal standards.

### Data analysis

Data were quantified and presented using Prism (version 5.01; GraphPad, USA). The lower limit of quantification (LLOQ) for each analyte was regarded as any peak with a signal-to-noise ratio greater than 4:1. Data are expressed as mean ± SEM.

### Validation

Validation was undertaken for 30 of the lipids that were representative of the majority of eicosanoids and related species. Because all the analytes are potentially found endogenously there is no appropriate “blank” matrix available for the validation. Therefore, all the analytes were spiked at known concentrations into equivalent 100 mg portions of homogenized “standard” rat brain tissue (combined from a number of animals) to determine the accuracy, precision, recovery, and ion suppression of the method using the method of standard addition as described previously (23, 28). This process ensured that the endogenous levels of all the analytes were equal between the samples used for validation. These endogenous levels were accounted for in all subsequent validation calculations.

Seven-point calibration curves spiked into rat brain tissue were used for each analyte at concentrations of 0.01–5 nmol/g. The 2-AG was spiked at concentrations of 0.1–50 nmol/g to account for the high endogenous levels of this analyte in rat brain. Linearity was calculated using the LC-MS/MS-determined peak area of each analyte expressed as a ratio to the peak area of the selected internal standard (chosen based on structural similarities).

Accuracy and precision values for intraday (n = 5) and interday (n = 4) were determined using rat brain tissue spiked with each analyte at concentrations of 0.02 nmol/g (low), 0.2 nmol/g (medium), and 0.8 nmol/g (high), except 2-AG which was spiked in at concentrations of 0.2, 2, and 8 nmol/g. Accuracy and precision values were calculated for each analyte from the peak areas and the relative standard deviation (RSD) of the replicates. Accuracy was determined as a ratio of the measured level of each analyte to the expected concentration. The LLOQ was the concentration at which the RSD of the analyte was found to be 20% or more. The limit of detection was the concentration at which the signal-to-noise ratio was greater than 3:1. Recovery values were calculated by comparing the peak area of each analyte at all three concentrations with the peak area of the equivalent 100% standard. Matrix effects were investigated by measuring ion suppression by spiking the analytes into the extracts from control (i.e., nonspiked) rat brain samples and comparing the peak area of each analyte to 100% standard equivalent where a 100% value represents the value for the absence of a sample matrix effect.

### Application of method to tissues from a rat monosodium iodoacetate model of OA pain

Intra-articular injection of monosodium iodoacetate (MIA) is a well-characterized and widely used rodent model of OA pain (29). Rats were anesthetized with isoflurane (3% in 50% N_2_O and 50% O_2_) before receiving an intra-articular injection of either MIA (1 mg/50 μl) in saline or 50 μl of saline (control) through the infra-patellar ligament of the left knee (30). Pain behavior was assessed for up to 28 days postinjection, as previously described (31). MIA-induced weight asymmetry was assessed using an incapacitance tester, and the changes in hind paw mechanical paw withdrawal thresholds were assessed using von Frey monofilaments (1–15 g), as previously described (31), and analyzed using Graphpad Prism 5 software. At day 28, rats were euthanized by stunning and decapitation, with tissue samples (spinal cord; brain divided into frontal cortex, midbrain, hippocampus, rest of cortex, and rest of brain regions; knee joints; and DRGs) dissected and extracted as described previously.

## RESULTS AND DISCUSSION

### Optimization of LC-MS/MS conditions

**Fig. 3.**
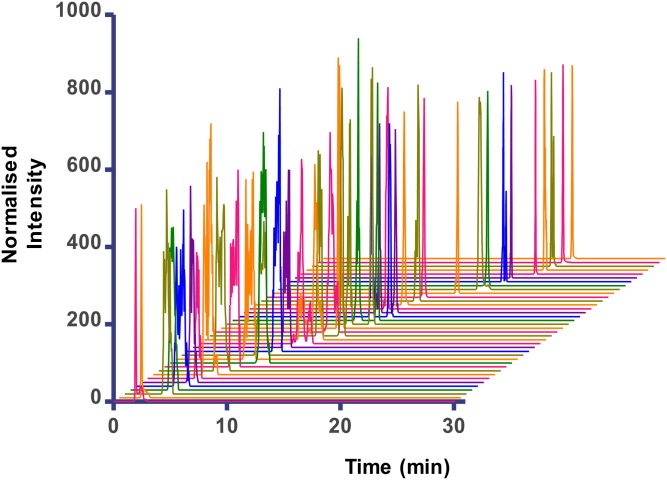
A 3D chromatogram of the individual separation of each analyte in order of elution time.

The pH and gradient elution profile of the mobile phases was optimized to completely separate the oxylipins into two groups, one that contained an ionizable carboxylic acid group (suitable for detection in electrospray negative ionization MS mode) and the other with a conjugated carboxylic acid group (suitable for detection in electrospray positive mode). This gradient profile allowed time for switching from negative ionization mode for the COOH-containing lipids to positive ionization mode for the later eluting conjugated lipids. An extracted ion LC-MS/MS chromatogram showing the separation of all 43 analytes and 6 internal standards (with the exception of PGD_2_ and PGE_2_) is shown in **Fig. 3**. Unique product ions for each analyte were used where possible to distinguish between analytes and to enable quantification by a combination of retention time and MRM transition (Table 1). The MRM transitions used were based on previous methods (23, 28), and further experimental optimization was conducted using product ion scans. No significant interfering peaks were found in the MRM channels of each analyte or internal standard, demonstrating that there was no “crosstalk” between these channels which might compromise selectivity, apart from the difficulty in distinguishing between PGD_2_ and PGE_2_, which is discussed below. Chromatographic separation of the structurally similar dihydroxyeicosatrienoic acids (DHETs), HETEs, and EETs was achieved; and where racemic mixtures of the lipids were used, these were resolved into two enantiomer peaks (for example *cis*- and *trans*-11-HETE; supplementary Fig. I). This fine tuning of separation is particularly important in a group of compounds in which many lipids are isobaric and where *cis* and *trans* isomers may vary in potency in their biological actions (21). In agreement with previous studies (32–34), we found that 5-HPETE and 12-HPETE were unstable and lost water in the MS source; and hence, we found that monitoring *m/z* 317 [M-H_2_O]^−^ as the precursor ion for these lipids produced a significant improvement in sensitivity compared with use of *m/z* 350 [M-H]^−^. The *m/z* 273 product ion was common to both the 5-HPETE and the 12-HPETE; hence, the unique product ions of *m/z* 153 for 12-HPETE (shown in supplementary Fig. II) and *m/z* 115 for 5-HPETE were selected to clearly distinguish these lipids.

PGD_2_, PGE_2_, and TXB_2_ had the same retention time in our method and also shared similar product ions. We used the unique MRM transition of *m/z* 369.23 > 169.09 for the detection of TXB_2_, which was apparent in both a standard spectra and in a rat brain sample extract (supplementary Fig. III). However, the product ion spectra of PGD_2_ and PGE_2_ were very similar and it proved difficult to find unique product ions. Consequently PGD_2_ and PGE_2_ could not be distinguished with confidence by the method.

### Validation of the LC-MS/MS method

The validation results in one tissue (rat brain) for 30 representative lipids by this method are shown in supplementary Table I. Linearity of the method was confirmed by a seven-point calibration curve of each analyte over a range of 0.01–5 nmol/g (and 0.1–50 nmol/g for 2-AG). The correlation coefficients (r^2^) in the validation ranged from 0.9311 to 0.9999 (supplementary Table I). Reproducible recoveries, generally greater than 50%, were observed for the majority of the analytes. The lipids 12-HPETE, 5-HPETE, 14,15-DHET, and AEA gave recovery values below 50%, but these recoveries were reproducible and did not affect the quantification of these lipids. Ion suppression analysis (supplementary Table I) indicated that the majority of the analytes were minimally affected by the rat brain tissue matrix, which agrees with a previous study in human blood plasma (23).

The accuracy and precision data for intraday (n = 5) and interday (n = 4) values for each analyte were within the recommended RSD of less than ±15%, except for two values discussed below. The intraday precision range was between 1.38 and 14.9% and the interday precision range was between 3.76 and 26.8%. The intraday accuracy range was between 89.2 and 114.3% and the interday precision range was between 85.2 and 111.4%. Both 19- and 20-HETE had poorer precision and accuracy values across all three concentrations compared with the other analytes. These fatty acids with terminal hydroxyl groups were poorly fragmented in electrospray MS, which has also been observed previously (22). Despite the instability and low recovery of 12-HPETE, it showed reasonable precision (RSD 15–27%) and accuracy (85–111%) values, hence demonstrating the method’s suitability for quantitative analysis of this lipid.

Overall, the developed analytical method was shown to be suitably validated to measure accurate and precise concentrations of oxylipins from a rat brain extract. The method was fully validated in brain tissue (relevant for other neural tissue such as spinal cord, DRGs, and brain regions), and for both rat blood plasma and knee tissue was shown to give reproducible and linear calibrations (data not shown). A typical example of an extracted ion chromatogram from the analysis of a spinal cord sample is shown in supplementary Fig. V, showing a profile of all the lipoxins measured in this individual sample.

### Tissue distribution of bioactive lipids in normal rats

**Fig. 4.**
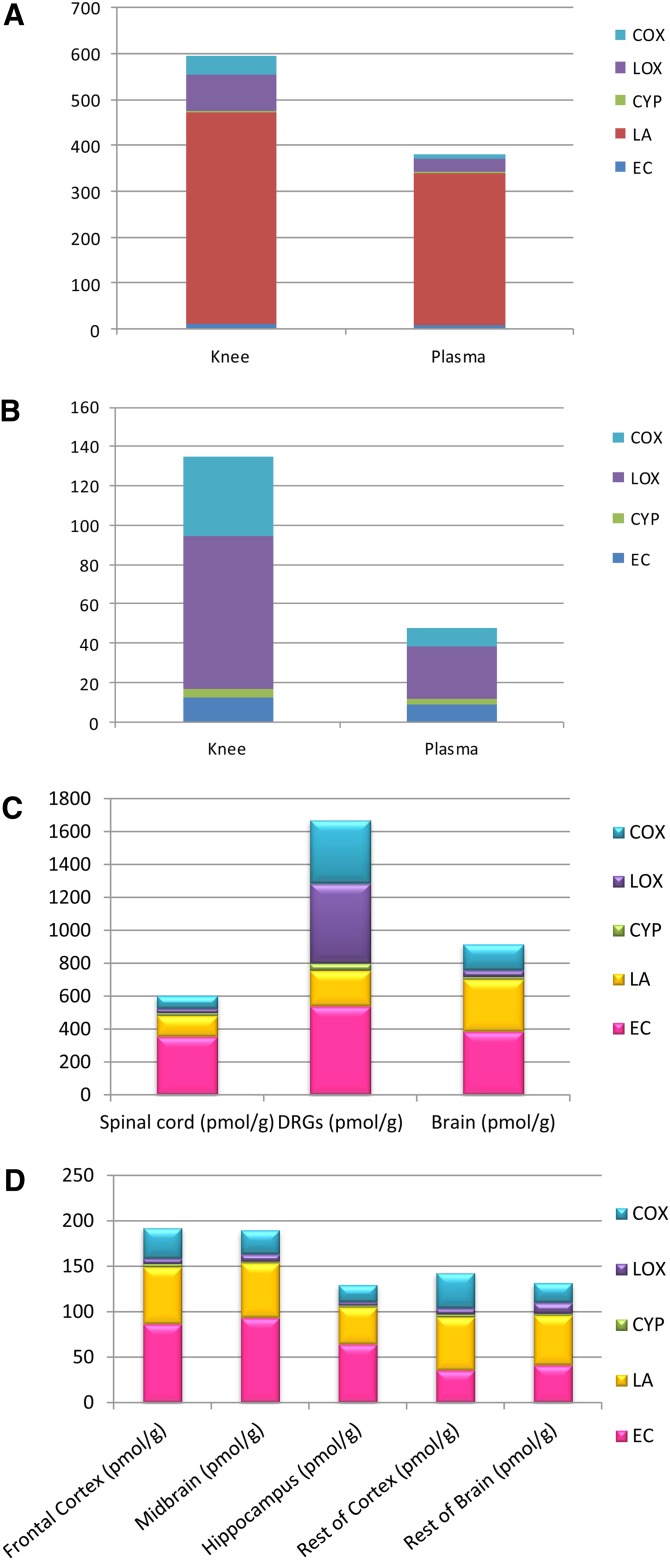
A: Lipid distribution of the measured analytes in knee joint (pmol/g) and plasma (pmol/ml), excluding 2-AG and AA. B: Lipid distribution of the measured analytes in knee joint and plasma, excluding LA, 2-AG, and AA. C: Lipid distribution of the measured analytes in spinal cord, DRGs, and brain. D: Lipid distribution of the measured analytes in rat brain divided into five regions; frontal cortex, midbrain, hippocampus, rest of cortex, and rest of brain

The method was used to generate a profile of the different bioactive lipids in a range of different tissues from naive rats. Eighteen lipids were measurable in these tissue samples: 5-HETE, 11-HETE, 12-HETE, 15-HETE, AEA, PEA, OEA, 2-AG, AraGly, PGD_2_/PGE_2_, TXB_2_, AA, LA, 9-HODE, and 13-HODE (**Fig. 4**, supplementary Table II). All other lipids listed in the method were below the LLOQ, and hence are not reported in figures or tables relating to the biological tissue analysis. In Fig. 4, the lipids are classified according to the enzyme responsible for their production (COX, LOX, or CYP) or as endocannabinoids and related compounds.

#### Blood plasma.

The range and concentration of oxylipins in rat plasma were broadly in agreement with previous studies with AA, LA, and 2-AG, constituting over 90% of the total oxylipins measured. Values for PGD_2_/PGE_2_, AA, and LA were somewhat lower than previously reported [supplementary Table II (35)], but those for AEA, PEA, and OEA (endocannabinoids) were in keeping with previous work: 3.1 ± 0.6 pmol/ml, 9.4 ± 1.6 pmol/ml, and 9.2 ± 1.8 pmol/ml, respectively (36). The 12-HETE has previously measured in rat plasma at levels around 100 ng/ml (37). Lipoxgenases are highly expressed in blood and immune cells (37, 38), which may account for the high levels of LA- and AA-derived LOX products in plasma: 9-HODE, 13-HODE, 9-oxoODE, 13-oxoODE, 12-HETE, and 15-HETE. The 12-HETE is primarily formed from the metabolism of AA by 12-LOX (Fig. 1), whereas the HODEs are formed through metabolism of LA by 15-LOX [for review see (6)]. The 15-LOX generates 15-HETE from AA. It is noteworthy that positional specificity of enzymes may be of pathophysiological importance because LOXs in the rat favor production of 12-HPETE, which is further metabolized to 12-HETE; whereas in humans, 15-HPETE is favored (39). Samples of blood plasma (and other tissues) were cooled, processed, and snap-frozen rapidly in an attempt to avoid known problems with artifactual generation of prostanoids due to platelet aggregation (40) or activation of phospholipases (41) and the generally lower values of PGD_2_/PGE_2_ reported here compared with previous literature values suggest that this was at least partially successful.

#### Knee joint.

Lipids in the rat knee joint are profiled here for the first time. A similar profile to that in rat plasma is reported, with AA (97%), LA (1%), and 2-AG (2%) comprising the majority of the lipids present. Levels of PGs in rabbit and human synovial fluid (42–44) and levels of endocannabinoids in human synovial fluid (45) have previously been reported. A higher proportion of LOX metabolites were present in the knee joint, compared with plasma. In addition, a high proportion of COX metabolites were also present in the knee joint, compared with plasma (Fig. 4)_._ The 9- and 13-oxoODE, oxidative metabolites of the HODEs, were only detectable in the knee joint and plasma (supplementary Table II). The level of LA detected in the knee joint was significantly higher than the other tissues; this is particularly evident when expressed as a ratio of AA.

#### DRGS and spinal cord.

The distribution of lipids within the DRGs and spinal cord (Fig. 4, supplementary Table II) was different to the distribution of lipids in plasma and knee joint. In the DRGs, 99% of the lipids were AA, with 2-AG making up 1%. The main difference when comparing DRGs to the spinal cord and brain was the high levels of LOX metabolites; however, the main precursor, LA, was not detectable in the DRGs. In the DRGs, 5-HETE, 11-HETE, 12-HETE, 15-HETE, AEA, PEA, OEA, 2-AG, PGD_2_/PGE_2_, TXB_2_, AA, LA, 9-HODE, and 13-HODE were all detected (Fig. 4, supplementary Table II). AraGly, along with 8-HETE, were both below the limit of quantification in this tissue. It is noteworthy that the LOX products (5-HETE and 12-HETE) can activate TRPV1, which is expressed by the small diameter sensory neurons, whose cell bodies are housed within the DRGs; hence, the presence of these bioactive lipids in the DRGs likely reflects a biological function. The 2-AG has previously been detected in the DRGs of the rat (46), however previously reported levels were considerably higher, which may reflect differences in extraction methods (46, 47). Comparison of the neural tissue revealed that levels of lipids in the DRGs are very high compared with the spinal cord and brain (Fig. 4, supplementary Table II).

In the spinal cord, the proportion of LA was relatively small and the major lipids were AA and 2-AG (supplementary Table II). Comparison of the distribution of the lipids between tissues revealed some interesting differences. In the spinal cord, 5-HETE, 11-HETE, 12-HETE, 15-HETE, AEA, PEA, OEA, 2-AG, AraGly, PGD_2_/PGE_2_, TXB_2_, AA, LA, 9-HODE, and 13-HODE were detected (supplementary Table II). The 8-HETE was below the limit of quantification and other lipids were not quantifiable in this tissue. These lipids have previously been measured in the spinal cord of rats, except for AraGly and 2-AG, using three separate methods: two LC-MS/MS methods (one positive mode and one negative mode) for analyzing the ethanolamines and eicosanoids, respectively, and a GS-MS method for analyzing the fatty acids (17). We and others have previously reported levels of 2-AG using a number of LC-MS/MS methods (46, 48–50), as well as AEA, OEA, and PEA (48) in the rat spinal cord. There is little information on spinal levels of eicosanoids apart from this publication; only PGD_2_ has previously been measured in mouse spinal cord (51).

In general, the levels of the analytes measured herein are much lower than previous reports (17, 48).

#### Brain regions.

Tissue was subdivided into five regions for analysis: frontal cortex, hippocampus, midbrain, rest of cortex, and rest of brain. In all brain regions, 5-HETE, 8-HETE, 11-HETE, 12-HETE, 15-HETE, AEA, PEA, OEA, 2-AG, AraGly, PGD_2_/PGE_2_, TXB_2_, AA, LA, 9-HODE, and 13-HODE were detected (Fig. 4, supplementary Table II). In the frontal cortex, AA made up 99% of the distribution, with 2-AG the remaining 1%; therefore, both these lipids were excluded again to show the distribution of the other lipids in this brain region. This was similar for the midbrain, hippocampus, rest of cortex, and rest of brain regions, where the AA made up 97–98% of the distribution with 2-AG making up the remainder. In whole rat cortex, 5-HETE, 8-HETE, 12-HETE, and 20-HETE have previously been measured (ranging from 2.6 to 12.14 pg/mg), and PGD_2_ and PGE_2_ have also been measured (1.76 and 3.97 pg/mg, respectively) (52). Previously, AA, PGD_2_, PGE_2,_ TXB_2_, 5-HETE, and 12-HETE were measured in ischemic rat brain; however, PGD_2_ and PGE_2_ were not detected in control brains (53). AEA, OEA, PEA, 2-AG, and AA have also been measured in the rat frontal cortex, where levels of these lipids were significantly higher than those reported with this method (54). AEA and 2-AG have been measured in the prefrontal cortex and hippocampus in rats (55). Endocannabinoids have also previously been measured in several different brain regions (28, 56, 57)

#### Regional differences in lipid profiles.

Comparison of the tissues revealed that the distribution of lipids in the spinal cord and brain are very similar (Fig. 4). The main difference between neural tissue and the knee and plasma is the relative amounts of AA versus LA. The knee joint and plasma had considerably more LA compared with AA; whereas, the converse was true for all brain regions. Interestingly, the DRGs and spinal cord had more equal levels of LA and AA present. A likely explanation for the high levels of AA in the brain is that neurons are not capable of AA synthesis from LA (58). Free AA is rapidly stored by esterification to membrane phospholipids (59, 60). Once released, AA can be metabolized by COX, LOX, or CYP to produce lipids which can modulate ion channels, pumps, protein kinases, and neurotransmitter uptake systems, and therefore neuronal activity.

**Fig. 5.**
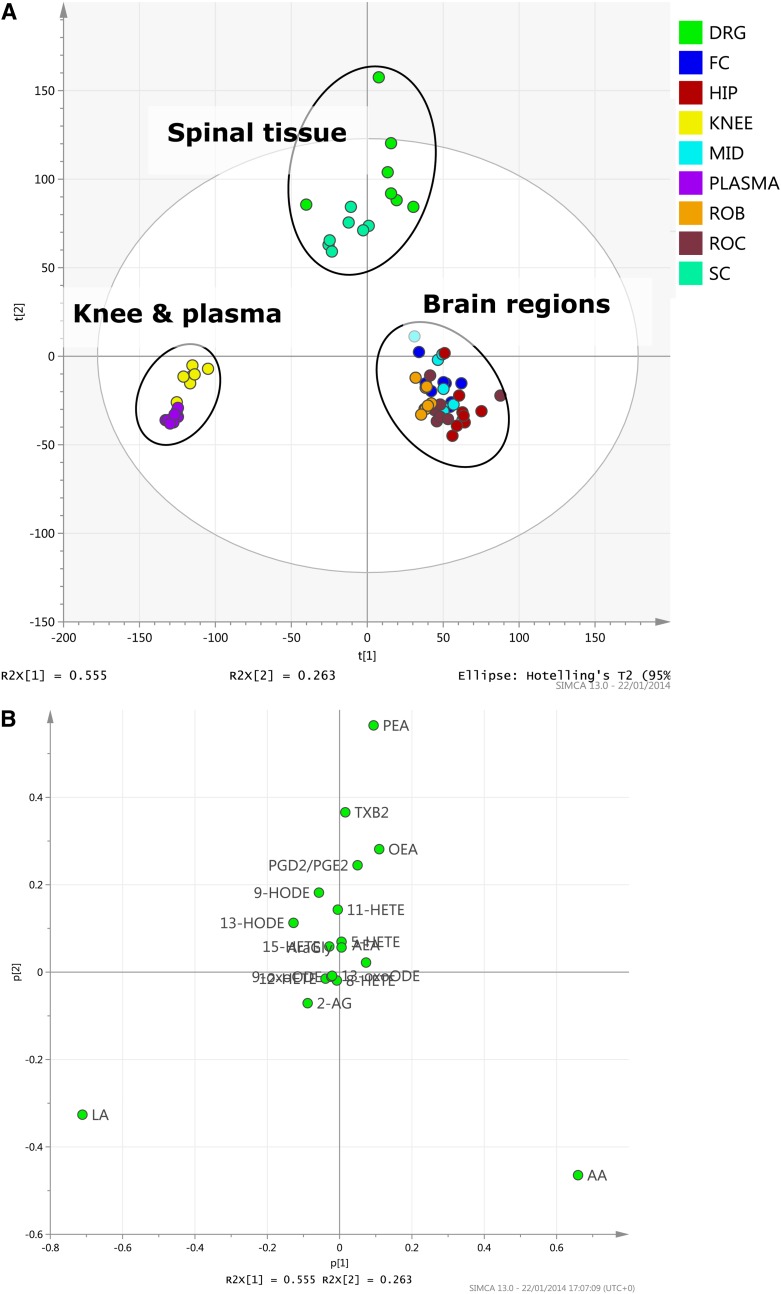
Lipid profiles from each rat tissue were analyzed using PCA to examine variation between samples represented by scores plot of a two-component PCA model of the dataset (A) and loadings plot of the same dataset (B). SC, spinal cord; FC, frontal cortex; HIP, hippocampus; MID, midbrain; ROC, rest of cortex; ROB, rest of brain.

Multivariate data analysis [principle component analysis (PCA)] of naive rat lipid profile data sets in all tissues (scores plot, **Fig. 5A**) emphasizes the usefulness of the analytical method to profile changes in the patterns of lipid distribution between tissues. PCA analysis shows three groups with similar distributions of oxylipins: *1*) plasma and knee, *2*) spinal cord and DRGs, and *3*) brain regions. The PCA loadings plot (Fig. 5B) shows the dominant lipids which typify these groups: LA higher in plasma and knee, PEA higher in spinal cord and DRGs, and AA higher in brain regions

### Profiling of bioactive lipid tissue distribution in a rat model of OA

Lipid profiles from saline- versus MIA-injected rats were compared to validate the use of this analytical method to determine changes in peripheral and central tissues under pathological conditions. Intra-articular injection of saline did not alter weight distribution (day 28 contralateral: ipsilateral difference = 2 ± 3 g) or hind paw withdrawal thresholds in the ipsilateral hind limb (day 28 = 15 g). Consistent with previous studies (30), intra-articular injection of MIA resulted in a significant decrease in weight bearing on the ipsilateral hind limb from day 7 onwards compared with saline-treated rats (day 28 contralateral: ipsilateral difference = 57 ± 12 g). In addition, intra-articular injection of MIA resulted in significant decreases in hind paw withdrawal thresholds to mechanical punctuate stimulation at day 28 (7 ± 2 g; *P* < 0.01) compared with saline-treated rats (15 ± 0 g).

**Fig. 6.**
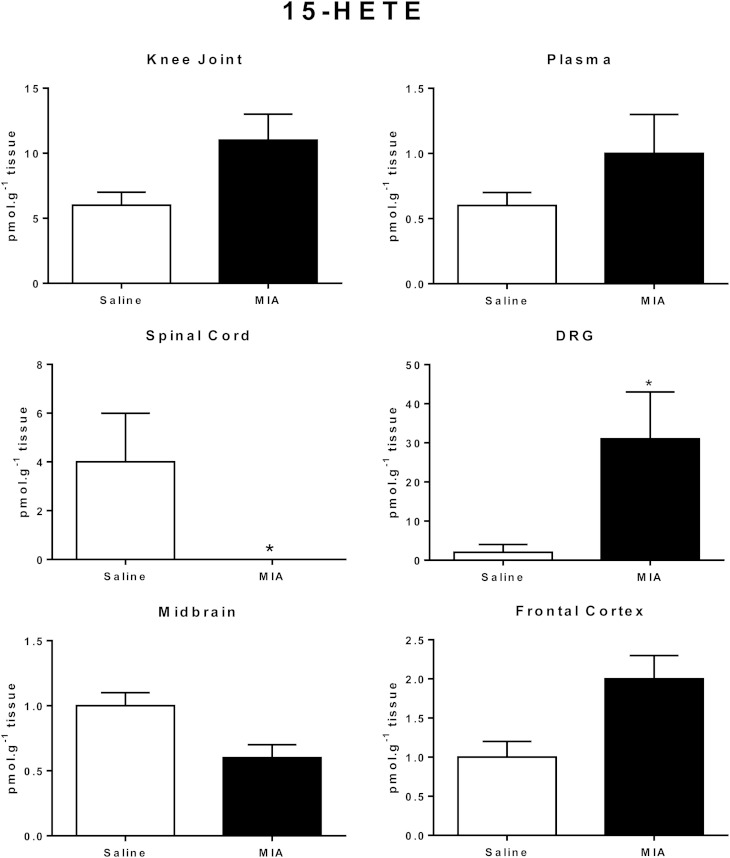
Levels of 15-HETE in knee joint, plasma, spinal cord, DRGs, midbrain. and frontal cortex.

On the whole, levels of the lipids did not vary significantly between MIA- and saline-treated rats (supplementary Table II). The exceptions were 12-HETE, which was significantly increased in the knee joint in MIA-treated rats compared with saline-treated rats, and 15-HETE, which was significantly increased in the DRGs in MIA-treated rats compared with saline-treated rats (supplementary Table II). Comparison of 15-HETE levels in the various tissues from saline- and MIA-treated rats revealed that the elevation of 15-HETE was tissue specific and not a generalized effect (**Fig. 6**). It is noteworthy that levels of 15-HETE were significantly decreased in the spinal cord of MIA-treated rats compared with saline-treated rats.

## CONCLUSIONS

The sensitive and selective LC-MS/MS method described allows comprehensive profiling of a wide range of bioactive lipids ranging from pro-inflammatory to anti-inflammatory oxylipins, providing a useful analytical tool for biological investigation. The use of polarity switching and careful manipulation of chromatographic separation has enabled simultaneous analysis of a structurally diverse range of compounds and provides a more representative picture of the in vivo inflammatory process.

A wide range of samples have been analyzed using this validated method which includes rat brain, spinal cord, knee joint, DRGs, plasma, hind paw, mouse brain and colon, and human plasma and colon. This has provided valuable information on the distribution of lipids in different tissues in relation to their biological function and involvement in biological processes. Alongside this, the method has been used to investigate changes in levels of bioactive lipids in a rat model of a disease state (OA) and also monitored for changes in these lipids following drug treatment. Additionally, the method can be used to investigate ligands of receptor targets such as TRPV1 and PPARs and to explore the significance of these targets in different disease models. To conclude, this LC-MS/MS method is a valuable analytical tool that can be applied to a diverse range of tissues, disease states, and biological targets.

## Supplementary Material

Supplemental Data

## References

[1] Rouzer C. A. Marnett L. J. 2011 Endocannabinoid oxygenation by cyclooxygenases, lipoxygenases, and cytochromes P450: cross-talk between the eicosanoid and endocannabinoid signaling pathways. Chem. Rev. 111: 5899–5921. 21923193 10.1021/cr2002799 PMC3191732

[2] Funk C. D. 2001 Prostaglandins and leukotrienes: advances in eicosanoid biology. Science. 294: 1871–1875. 11729303 10.1126/science.294.5548.1871

[3] Harizi H. Corcuff J. B. Gualde N. 2008 Arachidonic-acid-derived eicosanoids: roles in biology and immunopathology. Trends Mol. Med. 14: 461–469. 18774339 10.1016/j.molmed.2008.08.005

[4] Chopade A. R. Mulla W. A. 2010 Novel strategies for the treatment of inflammatory hyperalgesia. Eur. J. Clin. Pharmacol. 66: 429–444. 20155257 10.1007/s00228-010-0784-7

[5] Tapiero H. Ba G. N. Couvreur P. Tew K. D. 2002 Polyunsaturated fatty acids (PUFA) and eicosanoids in human health and pathologies. Biomed. Pharmacother. 56: 215–222. 12199620 10.1016/s0753-3322(02)00193-2

[6] Vangaveti V. Baune B. T. Kennedy R. L. 2010 Hydroxyoctadecadienoic acids: novel regulators of macrophage differentiation and atherogenesis. Ther. Adv. Endocrinol. Metab. 1: 51–60. 23148150 10.1177/2042018810375656 PMC3475286

[7] Hohmann A. G. Suplita R. L. 2nd 2006 Endocannabinoid mechanisms of pain modulation. AAPS J. 8: E693–E708. 17233533 10.1208/aapsj080479 PMC2751366

[8] Alexander S. P. Mathie A. Peters J. A. 2011 Guide to receptors and channels (GRAC), 5th edition. *Br. J. Pharmacol.* **164(Suppl 1):** S1–S324. 10.1111/j.1476-5381.2011.01649_1.x PMC3315626 22040146

[9] Alsalem M. Wong A. Millns P. Arya P. H. Chan M. S. Bennett A. Barrett D. A. Chapman V. Kendall D. A. 2013 The contribution of the endogenous TRPV1 ligands 9-HODE and 13-HODE to nociceptive processing and their role in peripheral inflammatory pain mechanisms. Br. J. Pharmacol. 168: 1961–1974. 23278358 10.1111/bph.12092 PMC3623065

[10] De Petrocellis L. Schiano Moriello A. Imperatore R. Cristino L. Starowicz K. Di Marzo V. 2012 A re-evaluation of 9-HODE activity at TRPV1 channels in comparison with anandamide: enantioselectivity and effects at other TRP channels and in sensory neurons. Br. J. Pharmacol. 167: 1643–1651. 22861649 10.1111/j.1476-5381.2012.02122.x PMC3525867

[11] Hwang S. W. Cho H. Kwak J. Lee S. Y. Kang C. J. Jung J. Cho S. Min K. H. Suh Y. G. Kim D. 2000 Direct activation of capsaicin receptors by products of lipoxygenases: endogenous capsaicin-like substances. Proc. Natl. Acad. Sci. USA. 97: 6155–6160. 10823958 10.1073/pnas.97.11.6155 PMC18574

[12] Patwardhan A. M. Akopian A. N. Ruparel N. B. Diogenes A. Weintraub S. T. Uhlson C. Murphy R. C. Hargreaves K. M. 2010 Heat generates oxidized linoleic acid metabolites that activate TRPV1 and produce pain in rodents. J. Clin. Invest. 120: 1617–1626. 20424317 10.1172/JCI41678 PMC2860941

[13] Patwardhan A. M. Scotland P. E. Akopian A. N. Hargreaves K. M. 2009 Activation of TRPV1 in the spinal cord by oxidized linoleic acid metabolites contributes to inflammatory hyperalgesia. Proc. Natl. Acad. Sci. USA. 106: 18820–18824. 19843694 10.1073/pnas.0905415106 PMC2764734

[14] Julius D. 2013 TRP channels and pain. Annu. Rev. Cell Dev. Biol. 29: 355–384. 24099085 10.1146/annurev-cellbio-101011-155833

[15] Nithipatikom K. Grall A. J. Holmes B. B. Harder D. R. Falck J. R. Campbell W. B. 2001 Liquid chromatographic-electrospray ionization-mass spectrometric analysis of cytochrome P450 metabolites of arachidonic acid. Anal. Biochem. 298: 327–336. 11700990 10.1006/abio.2001.5395

[16] Yoshida Y. Kodai S. Takemura S. Minamiyama Y. Niki E. 2008 Simultaneous measurement of F2-isoprostane, hydroxyoctadecadienoic acid, hydroxyeicosatetraenoic acid, and hydroxycholesterols from physiological samples. Anal. Biochem. 379: 105–115. 18482573 10.1016/j.ab.2008.04.028

[17] Buczynski M. W. Svensson C. I. Dumlao D. S. Fitzsimmons B. L. Shim J. H. Scherbart T. J. Jacobsen F. E. Hua X. Y. Yaksh T. L. Dennis E. A. 2010 Inflammatory hyperalgesia induces essential bioactive lipid production in the spinal cord. J. Neurochem. 114: 981–993. 20492349 10.1111/j.1471-4159.2010.06815.x PMC3994888

[18] Dumlao D. S. Buczynski M. W. Norris P. C. Harkewicz R. Dennis E. A. 2011 High-throughput lipidomic analysis of fatty acid derived eicosanoids and N-acylethanolamines. Biochim. Biophys. Acta. **1811:** 724–736. 21689782 10.1016/j.bbalip.2011.06.005 PMC3205334

[19] Masoodi M. Mir A. A. Petasis N. A. Serhan C. N. Nicolaou A. 2008 Simultaneous lipidomic analysis of three families of bioactive lipid mediators leukotrienes, resolvins, protectins and related hydroxy-fatty acids by liquid chromatography/electrospray ionisation tandem mass spectrometry. Rapid Commun. Mass Spectrom. 22: 75–83. 18059001 10.1002/rcm.3331 PMC2542421

[20] Yang J. Schmelzer K. Georgi K. Hammock B. D. 2009 Quantitative profiling method for oxylipin metabolome by liquid chromatography electrospray ionization tandem mass spectrometry. Anal. Chem. 81: 8085–8093. 19715299 10.1021/ac901282n PMC3290520

[21] Jiang H. McGiff J. C. Quilley J. Sacerdoti D. Reddy L. M. Falck J. R. Zhang F. Lerea K. M. Wong P. Y. 2004 Identification of 5,6-trans-epoxyeicosatrienoic acid in the phospholipids of red blood cells. J. Biol. Chem. 279: 36412–36418. 15213230 10.1074/jbc.M403962200

[22] Masoodi M. Eiden M. Koulman A. Spaner D. Volmer D. A. 2010 Comprehensive lipidomics analysis of bioactive lipids in complex regulatory networks. Anal. Chem. 82: 8176–8185. 20828216 10.1021/ac1015563

[23] Zhang J. H. Pearson T. Matharoo-Ball B. Ortori C. A. Warren A. Y. Khan R. Barrett D. A. 2007 Quantitative profiling of epoxyeicosatrienoic, hydroxyeicosatetraenoic, and dihydroxyeicosatetraenoic acids in human intrauterine tissues using liquid chromatography/electrospray ionization tandem mass spectrometry. Anal. Biochem. 365: 40–51. 17418798 10.1016/j.ab.2007.03.001

[24] Kingsley P. J. Rouzer C. A. Saleh S. Marnett L. J. 2005 Simultaneous analysis of prostaglandin glyceryl esters and prostaglandins by electrospray tandem mass spectrometry. Anal. Biochem. 343: 203–211. 16004953 10.1016/j.ab.2005.05.005

[25] Caraceni P. Viola A. Piscitelli F. Giannone F. Berzigotti A. Cescon M. Domenicali M. Petrosino S. Giampalma E. Riili A. 2010 Circulating and hepatic endocannabinoids and endocannabinoid-related molecules in patients with cirrhosis. Liver Int. 30: 816–825. 19840245 10.1111/j.1478-3231.2009.02137.x

[26] Kilaru A. Isaac G. Tamura P. Baxter D. Duncan S. R. Venables B. J. Welti R. Koulen P. Chapman K. D. 2010 Lipid profiling reveals tissue-specific differences for ethanolamide lipids in mice lacking fatty acid amide hydrolase. Lipids. 45: 863–875. 20714818 10.1007/s11745-010-3457-5 PMC2944412

[27] Lehtonen M. Storvik M. Malinen H. Hyytia P. Lakso M. Auriola S. Wong G. Callaway J. C. 2011 Determination of endocannabinoids in nematodes and human brain tissue by liquid chromatography electrospray ionization tandem mass spectrometry. J. Chromatogr. B Analyt. Technol. Biomed. Life Sci. 879: 677–694. 10.1016/j.jchromb.2011.02.004 21367677

[28] Richardson D. Ortori C. A. Chapman V. Kendall D. A. Barrett D. A. 2007 Quantitative profiling of endocannabinoids and related compounds in rat brain using liquid chromatography-tandem electrospray ionization mass spectrometry. Anal. Biochem. 360: 216–226. 17141174 10.1016/j.ab.2006.10.039

[29] Sagar D. R. Suokas A. K. Walsh D. A. Chapman V. 2013 Translational relevance of animal models of osteoarthritic pain. *In* Pain Models: Translational Relevance and Applications. H. O. Handwerker and L. Arendt-Nielsen, editors. IASP Press, Washington DC.

[30] Sagar D. R. Staniaszek L. E. Okine B. N. Woodhams S. Norris L. M. Pearson R. G. Garle M. J. Alexander S. P. H. Bennett A. J. Barrett D. A. 2010 Tonic modulation of spinal hyperexcitability by the endocannabinoid receptor system in a rat model of osteoarthritis pain. Arthritis Rheum. 62: 3666–3676. 20722027 10.1002/art.27698 PMC3132591

[31] Sagar D. R. Burston J. J. Hathway G. J. Woodhams S. G. Pearson R. G. Bennett A. J. Kendall D. A. Scammell B. E. Chapman V. 2011 The contribution of spinal glial cells to chronic pain behaviour in the monosodium iodoacetate model of osteoarthritic pain. Mol. Pain. 7: 88. 22093915 10.1186/1744-8069-7-88 PMC3271989

[32] Garscha U. Nilsson T. Oliw E. H. 2008 Enantiomeric separation and analysis of unsaturated hydroperoxy fatty acids by chiral column chromatography-mass spectrometry. J. Chromatogr. B Analyt. Technol. Biomed. Life Sci. 872: 90–98. 10.1016/j.jchromb.2008.07.013 18667369

[33] Havrilla C. M. Hachey D. L. Porter N. A. 2000 Coordination (Ag+) ion spray-mass spectrometry of peroxidation products of cholesterol linoleate and cholesterol arachidonate: high-performance liquid chromatography-mass spectrometry analysis of peroxide products from polyunsaturated lipid autoxidation. J. Am. Chem. Soc. 122: 8042–8055.

[34] MacMillan D. K. Murphy R. C. 1995 Analysis of lipid hydroperoxides and long-chain conjugated keto acids by negative ion electrospray mass spectrometry. J. Am. Soc. Mass Spectrom. 6: 1190–1201. 24214070 10.1016/1044-0305(95)00505-6

[35] Bai Y. J. Gao X. Y. Lu J. Q. Zhang H. G. 2010 A LC-MS-based method for quantification of biomarkers from serum of allergic rats. Molecules. 15: 3356–3365. 20657485 10.3390/molecules15053356 PMC6263330

[36] Giuffrida A. Rodriguez de Fonseca F. Piomelli D. 2000 Quantification of bioactive acylethanolamides in rat plasma by electrospray mass spectrometry. Anal. Biochem. 280: 87–93. 10805525 10.1006/abio.2000.4509

[37] Yamamoto S. Takahashi Y. Hada T. Hagiya H. Suzuki H. Reddy G. R. Ueda N. Arakawa T. Nakamura M. Matsuda S. 1997 Mammalian arachidonate 12-lipoxygenases. Adv. Exp. Med. Biol. 400A: 127–131. 9547547 10.1007/978-1-4615-5325-0_18

[38] Yoshimoto T. Takahashi Y. 2002 Arachidonate 12-lipoxygenases. Prostaglandins Other Lipid Mediat. 68–69: 245–262. 10.1016/s0090-6980(02)00034-5 12432922

[39] Kühn H. O’Donnell V. B. 2006 Inflammation and immune regulation by 12/15-lipoxygenases. Prog. Lipid Res. 45: 334–356. 16678271 10.1016/j.plipres.2006.02.003

[40] Smyth E. M. Grosser T. Wang M. Yu Y. FitzGerald G. A. 2009 Prostanoids in health and disease. J. Lipid Res. 50(Suppl): S423–S428. 19095631 10.1194/jlr.R800094-JLR200 PMC2674745

[41] Tithof P. K. Roberts M. P. Guan W. Elgayyar M. Godkin J. D. 2007 Distinct phospholipase A2 enzymes regulate prostaglandin E2 and F2alpha production by bovine endometrial epithelial cells. Reprod. Biol. Endocrinol. 5: 16. 17459165 10.1186/1477-7827-5-16 PMC1868772

[42] Alstergren P. Kopp S. 2000 Prostaglandin E2 in temporomandibular joint synovial fluid and its relation to pain and inflammatory disorders. J. Oral Maxillofac. Surg. 58: 180–186, discussion 186–188. 10670597 10.1016/s0278-2391(00)90335-5

[43] Gold E. W. Anderson L. B. Schwartz E. R. Miller C. W. 1976 The effect of salicylate on prostaglandin levels in rabbit knees following inducement of osteoarthritic changes. Prostaglandins. 12: 837–842. 981706 10.1016/0090-6980(76)90057-5

[44] Schumacher H. R. Jr Meng Z. Sieck M. Zonay L. Clayburne G. Baker J. F. Park J. Baker D. G. 1996 Effect of a nonsteroidal antiinflammatory drug on synovial fluid in osteoarthritis. J. Rheumatol. 23: 1774–1777. 8895157

[45] Richardson D. Pearson R. G. Kurian N. Latif M. L. Garle M. J. Barrett D. A. Kendall D. A. Scammell B. E. Reeve A. J. Chapman V. 2008 Characterisation of the cannabinoid receptor system in synovial tissue and fluid in patients with osteoarthritis and rheumatoid arthritis. Arthritis Res. Ther. 10: R43. 18416822 10.1186/ar2401 PMC2453762

[46] Huang S. M. Strangman N. M. Walker J. M. 1999 Liquid chromatographic-mass spectrometric measurement of the endogenous cannabinoid 2-arachidonylglycerol in the spinal cord and peripheral nervous system. Zhongguo Yao Li Xue Bao. 20: 1098–1102. 11189199

[47] Mitrirattanakul S. Ramakul N. Guerrero A. V. Matsuka Y. Ono T. Iwase H. Mackie K. Faull K. F. Spigelman I. 2006 Site-specific increases in peripheral cannabinoid receptors and their endogenous ligands in a model of neuropathic pain. Pain. 126: 102–114. 16844297 10.1016/j.pain.2006.06.016 PMC1776167

[48] Jhaveri M. D. Richardson D. Kendall D. A. Barrett D. A. Chapman V. 2006 Analgesic effects of fatty acid amide hydrolase inhibition in a rat model of neuropathic pain. J. Neurosci. 26: 13318–13327. 17182782 10.1523/JNEUROSCI.3326-06.2006 PMC6674985

[49] Petrosino S. Palazzo E. de Novellis V. Bisogno T. Rossi F. Maione S. Di Marzo V. 2007 Changes in spinal and supraspinal endocannabinoid levels in neuropathic rats. Neuropharmacology. 52: 415–422. 17011598 10.1016/j.neuropharm.2006.08.011

[50] Staniaszek L. E. Norris L. M. Kendall D. A. Barrett D. A. Chapman V. 2010 Effects of COX-2 inhibition on spinal nociception: the role of endocannabinoids. Br. J. Pharmacol. 160: 669–676. 20590570 10.1111/j.1476-5381.2010.00703.x PMC2931566

[51] Kihara Y. Matsushita T. Kita Y. Uematsu S. Akira S. Kira J. Ishii S. Shimizu T. 2009 Targeted lipidomics reveals mPGES-1–PGE2 as a therapeutic target for multiple sclerosis. Proc. Natl. Acad. Sci. USA. 106: 21807–21812. 19995978 10.1073/pnas.0906891106 PMC2789753

[52] Yue H. Jansen S. A. Strauss K. I. Borenstein M. R. Barbe M. F. Rossi L. J. Murphy E. 2007 A liquid chromatography/mass spectrometric method for simultaneous analysis of arachidonic acid and its endogenous eicosanoid metabolites prostaglandins, dihydroxyeicosatrienoic acids, hydroxyeicosatetraenoic acids, and epoxyeicosatrienoic acids in rat brain tissue. J. Pharm. Biomed. Anal. 43: 1122–1134. 17125954 10.1016/j.jpba.2006.10.009 PMC2855500

[53] Farias S. E. Basselin M. Chang L. Heidenreich K. A. Rapoport S. I. Murphy R. C. 2008 Formation of eicosanoids, E2/D2 isoprostanes, and docosanoids following decapitation-induced ischemia, measured in high-energy-microwaved rat brain. J. Lipid Res. 49: 1990–2000. 18503030 10.1194/jlr.M800200-JLR200 PMC2515526

[54] Williams J. Wood J. Pandarinathan L. Karanian D. A. Bahr B. A. Vouros P. Makriyannis A. 2007 Quantitative method for the profiling of the endocannabinoid metabolome by LC-atmospheric pressure chemical ionization-MS. Anal. Chem. 79: 5582–5593. 17600384 10.1021/ac0624086

[55] Malinen H. Lehtonen M. Hyytia P. 2009 Modulation of brain endocannabinoid levels by voluntary alcohol consumption in alcohol-preferring AA rats. Alcohol. Clin. Exp. Res. 33: 1711–1720. 19572983 10.1111/j.1530-0277.2009.01008.x

[56] Buczynski M. W. Parsons L. H. 2010 Quantification of brain endocannabinoid levels: methods, interpretations and pitfalls. Br. J. Pharmacol. 160: 423–442. 20590555 10.1111/j.1476-5381.2010.00787.x PMC2931546

[57] Stuart J. M. Paris J. J. Frye C. Bradshaw H. B. 2013 Brain levels of prostaglandins, endocannabinoids, and related lipids are affected by mating strategies. Int. J. Endocrinol. 2013: 436252. 24369463 10.1155/2013/436252 PMC3863470

[58] DeMar J. C. Jr Lee H. J. Ma K. Chang L. Bell J. M. Rapoport S. I. Bazinet R. P. 2006 2006. Brain elongation of linoleic acid is a negligible source of the arachidonate in brain phospholipids of adult rats. Biochim. Biophys. Acta. **1761:** 1050–1059. 16920015 10.1016/j.bbalip.2006.06.006

[59] Piomelli D. 1993 Arachidonic acid in cell signaling. Curr. Opin. Cell Biol. 5: 274–280. 7685181 10.1016/0955-0674(93)90116-8

[60] Piomelli D. 1994 Eicosanoids in synaptic transmission. Crit. Rev. Neurobiol. 8: 65–83. 8124731

