# Supplementary material for: Simultaneous tissue profiling of eicosanoid and endocannabinoid lipid families in a rat model of osteoarthritis
Source: J Lipid Res. 2014 Sep;55(9):1902–13. doi: 10.1194/jlr.M048694 (PMC4617365; doi:10.1194/jlr.M048694)
Supplement: Supplemental Data [file supp_55_9_1902__index.html]

Simultaneous tissue profiling of eicosanoid and endocannabinoid lipid families in a rat model of osteoarthritis — Simultaneous tissue profiling of eicosanoid and endocannabinoid lipid families in a rat model of osteoarthritis — Supplemental Data 

# Simultaneous tissue profiling of eicosanoid and endocannabinoid lipid families in a rat model of osteoarthritis

## Supplemental Data

**Files in this Data Supplement:**

- Supplementary information - Supplementary Information
