# Supplementary material for: Simultaneous tissue profiling of eicosanoid and endocannabinoid lipid families in a rat model of osteoarthritis
Source: J Lipid Res. 2014 Sep;55(9):1902–13. doi: 10.1194/jlr.M048694 (PMC4617365; doi:10.1194/jlr.M048694)
Supplement: Supplemental Data [file supp_M048694_jlr.M048694-1.pdf]

SUPPLEMENTARY INFORMATION

Simultaneous tissue profiling of eicosanoid and endocannabinoid lipid families in a rat model of osteoarthritis

A. Wong*[1], D.R. Sagar*[2] C.A. Ortori[1], D.A. Kendall[2], V. Chapman[2], D.A. Barrett[1].

*Joint first authors

Centre for Analytical Bioscience, School of Pharmacy[1], School of Life Sciences[2], University of Nottingham, Nottingham, NG7 2UH, UK

Corresponding author:

DA Barrett

telephone +44(0)1159515062

fax +44 (0)1159515102

david.barrett@nottingham.ac.uk

List of oxylipins standards used in the method

The following lipids were quantified: prostaglandin D_2 ethanolamide (PGD_2-EA), prostaglandin E_1 ethanolamide (PGE_1-EA), prostaglandin E_2 ethanolamide (PGE_2-EA), prostaglandin $F_{2\alpha}$ ethanolamide ($PGF_{2\alpha}$-EA), 5,6-dihydroxyeicosatrienoic acid (5,6-DHET), 8,9-dihydroxyeicosatrienoic acid (8,9-DHET), 11,12-dihydroxyeicosatrienoic acid (11,12-DHET), 14,15-dihydroxyeicosatrienoic acid (14,15-DHET), 8,15-dihydroxyeicosatetraenoic acid (8,15-DiHETE), 9-hydroxyeicosatetraenoic acid (9-HETE), 11-hydroxyeicosatetraenoic acid (11-HETE), 12-hydroxyeicosatetraenoic acid (12-HETE), 15-hydroxyeicosatetraenoic acid (15-HETE), 16-hydroxyeicosatetraenoic acid (16-HETE), 19-hydroxyeicosatetraenoic acid (19-HETE), 20-hydroxyeicosatetraenoic acid (20-HETE), arachidonic acid (AA), linoleic acid (LA), 5-hydroperoxyeicosatetraenoic acid (5-HPETE), 9-hydroxyoctadecadienoic acid (9-HODE), 13-hydroxyoctadecadienoic acid (13-HODE), 9-oxooctadecadienoic acid (9-oxoODE), 13-oxooctadecadienoic acid (13-oxoODE), leukotriene-B_4 (LTB_4), leukotriene-E_4 (LTE_4), prostaglandin D_2 (PGD_2), prostaglandin E_2 (PGE_2), thromboxane-B_2 (TXB_2), resolvin D1 (RvD1), resolvin D2 (RvD2), N-arachidonoyl ethanolamide (AEA), 2-arachidonoyl glycerol (2-AG), N-palmitoyl ethanolamide (PEA), N-oleoyl ethanolamide (OEA), N-arachidonoyl dopamine (NADA), N-arachidonoyl glycine (AraGly), 5,6-epoxyeicosatrienamide (5,6-EET-EA), 2-(14,15-epoxyeicosatrienoyl)-glycerol (14,15-EET-G), arachidonic acid-d8 (AA-d8), prostaglandin D_2-d4 (PGD_2-d4), prostaglandin $F_{2\alpha}$ ethanolamide-d4 ($PGF_{2\alpha}$-EA-d4), N-arachidonoyl ethanolamide-d8 (AEA-d8) and 2-arachidonoyl glycerol-d8 (2-AG-d8) were purchased from Cambridge Bioscience (Cambridge, UK). 5-hydroxyeicosatetraenoic acid (5-HETE), 8-hydroxyeicosatetraenoic acid (8-HETE), 5,6-epoxyeicosatrienoic acid (5,6-EET), 8,9-epoxyeicosatrienoic acid (8,9-EET), 11,12-epoxyeicosatrienoic acid (11,12-EET), 14,15-epoxyeicosatrienoic acid (14,15-EET), 12-hydroperoxyeicosatetraenoic acid (12-HPETE) and 15-hydroxyeicosatetraenoic acid-d8 (15-HETE-d8) were all purchased from Biomol International (Exeter, UK). HPLC grade water (ELGA Ltd. High Wycombe, UK) was used

in all experiments. All stock solutions of each compound were diluted in ethanol. Serial dilutions of these were used for calibration.

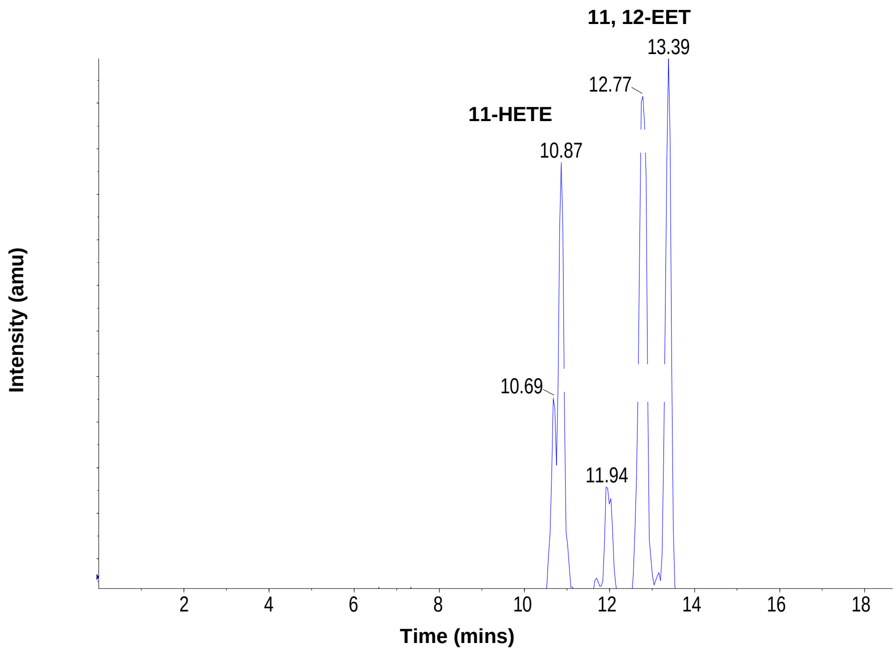

Supplementary Figure S1. LC-MS/MS separation of 11-HETE and 11,12-EET (both at *m/z* 319.24/167.11). There are two distinct peaks for 11,12-EET.

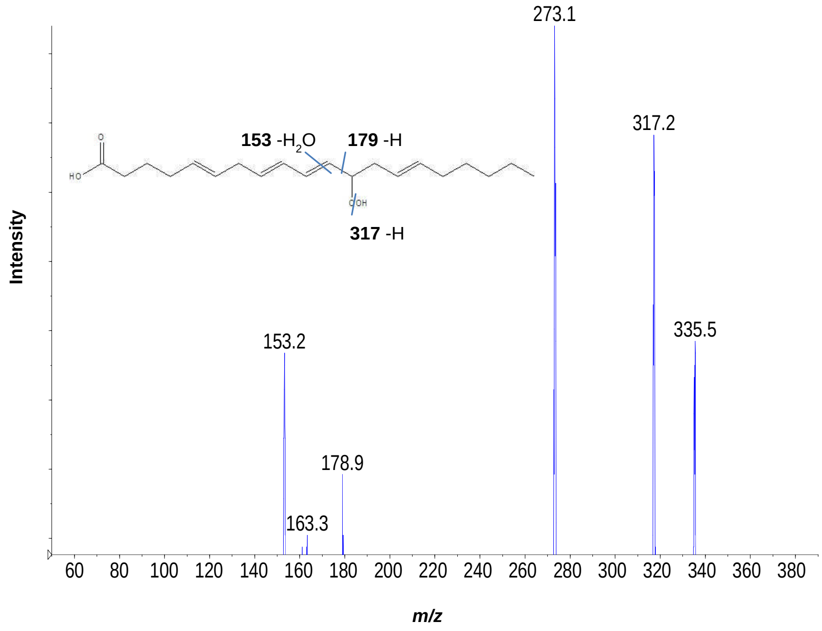

Supplementary Figure S2. Electrospray MS product ion spectrum of 12-HPETE, showing the fragmentation points on the structure of the lipid.

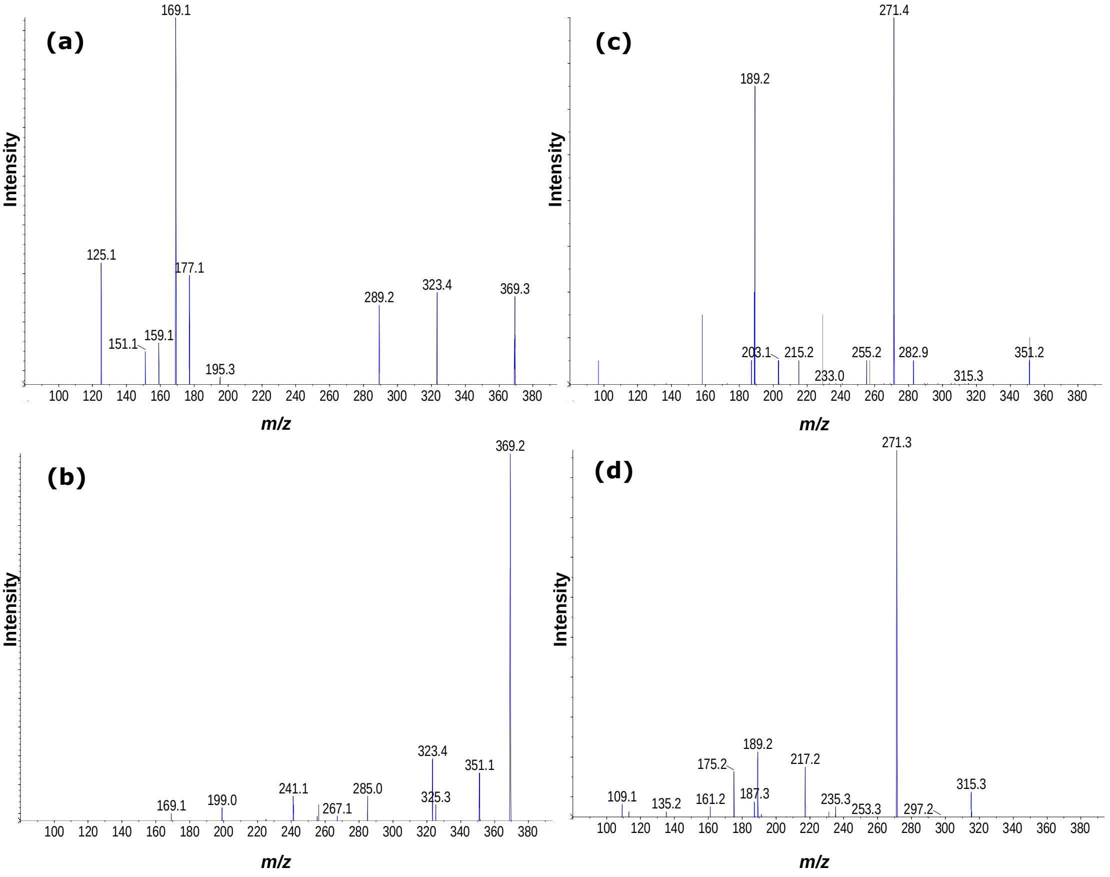

Supplementary Figure S3. Product ion electrospray MS spectra of (a) TXB_2 standard, (b) TXB_2 peak in a brain sample, (c) PGD_2 standard and (d) PGE_2 standard.

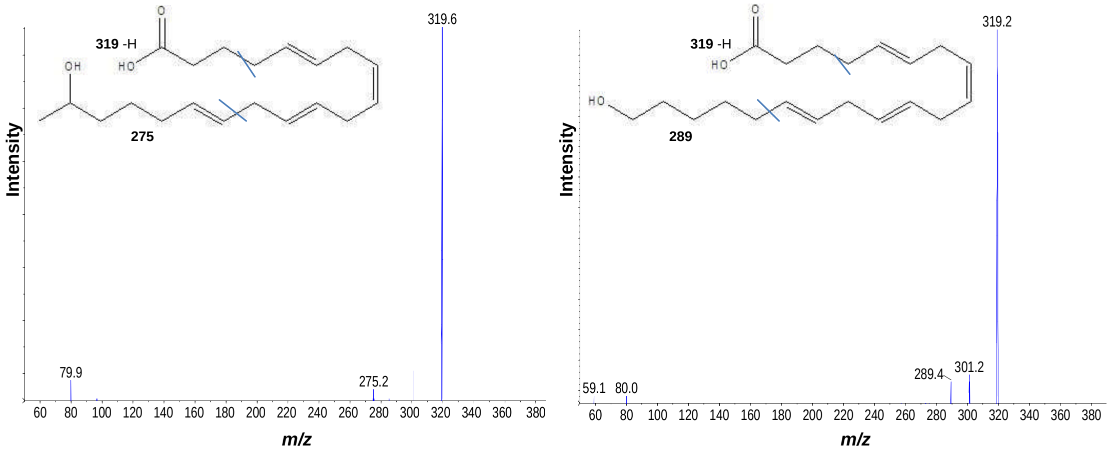

Supplementary Figure S4. Product ion spectra of 19- and 20-HETE and the fragmentation of both these lipids.

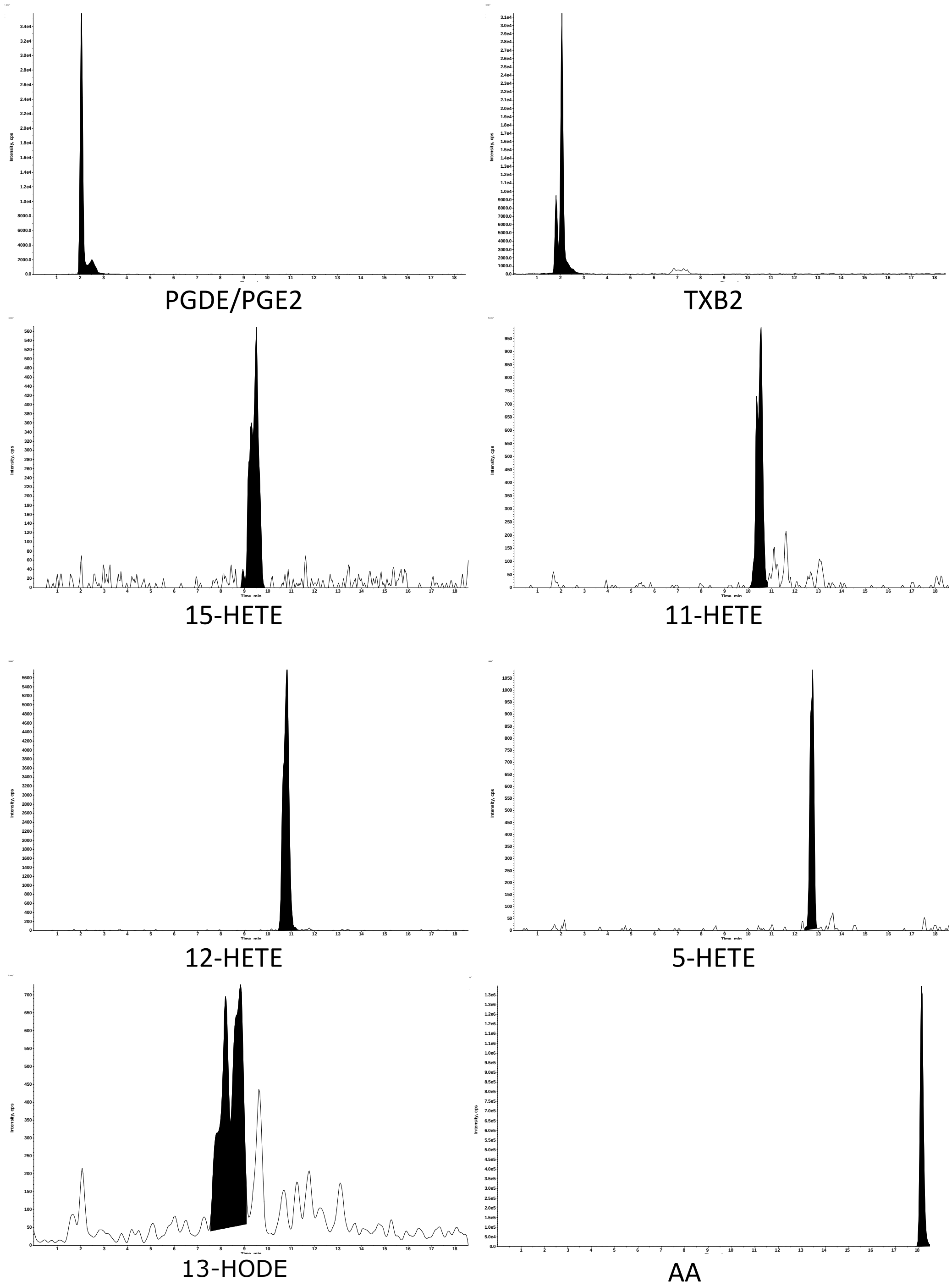

Supplementary Figure S5 A typical example showing extracted ion LC-MS/MS chromatograms from the analysis of an individual rat spinal cord sample showing a profile of all measurable lipoxins. The filled peak is the measured peak for each lipid. All in negative ion ESI mode apart from 2-AG, AEA, PEA and OEA which are in positive ion ESI mode.

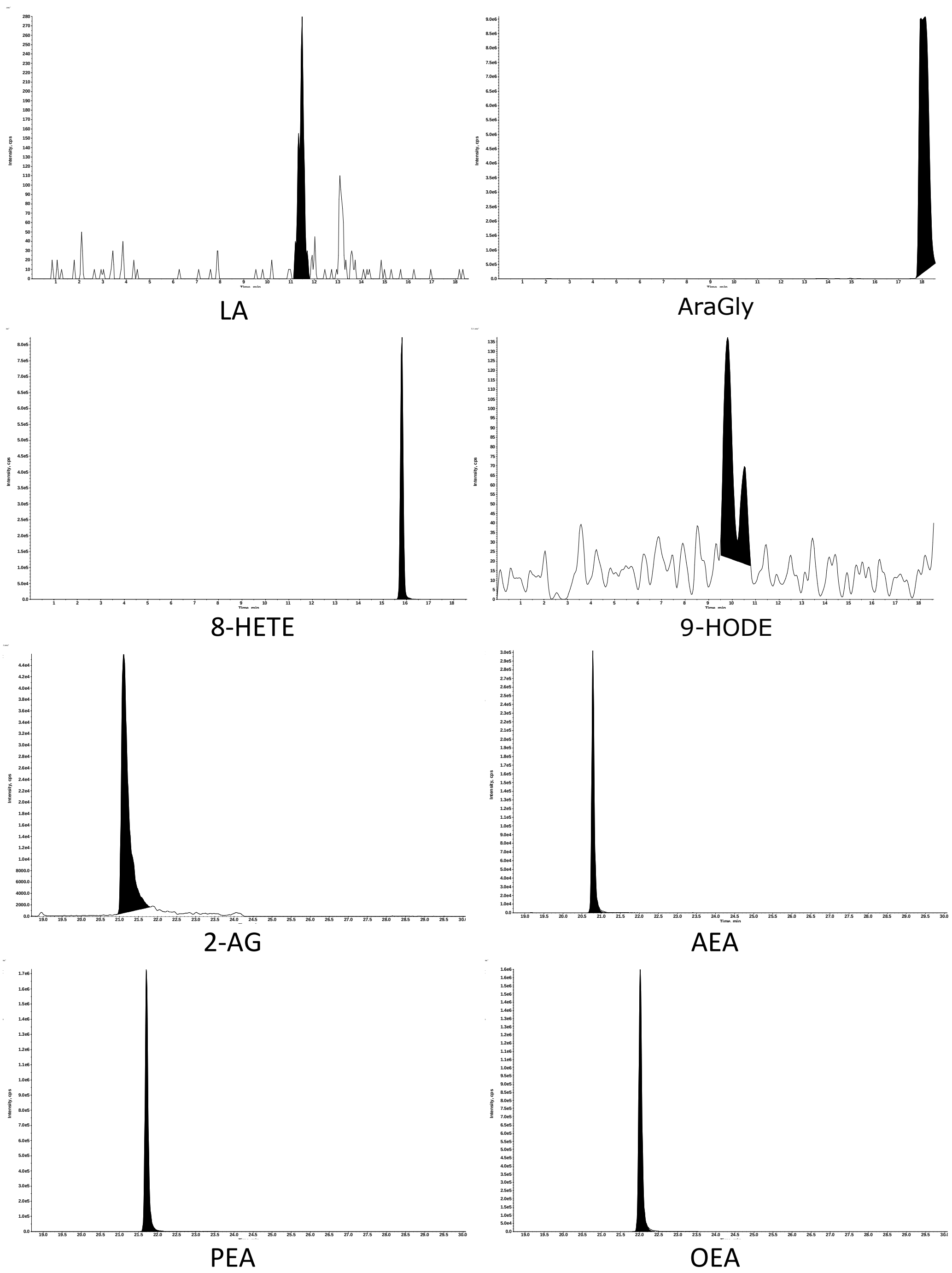

Supplementary Figure S5 (continued). A typical example showing extracted ion LC-MS/MS chromatograms from the analysis of an individual rat spinal cord sample showing a profile of all measurable lipoxins. The filled peak is the measured peak for each lipid. All in negative ion ESI mode apart from 2-AG, AEA, PEA and OEA which are in positive ion ESI mode.

Supplementary Table S1. Validation table of all 30 compounds investigated, n = 6 for accuracy and precision values for low, medium and high concentrations.

Analyte	R^2	Slope	Concentration (nmol/g)	Recovery (%)	ION SUPP (%)	Intraday (n = 5) Precision (RSD%)	Intraday (n = 5) Accuracy (RSD%)	Interday (n = 4) Precision (RSD%)	Interday (n = 4) Accuracy (RSD%)
**PGD2 EA**	0.9928	y=1.474x+0.1612	**0.02**	59.9 ± 3.1	61.2 ± 6.0	4.72	104.9	10.3	95.9
			**0.2**	49.6 ± 5.0	60.2 ± 7.3	9.41	103.0	9.16	101.7
			**0.8**	59.4 ± 4.7	73.8 ± 4.2	1.38	100.9	4.67	102.8
**PGE1 EA**	0.9925	y=15.19x+2.0886	**0.02**	58.6 ± 3.7	49.7 ± 2.9	3.33	92.0	13.7	85.8
			**0.2**	60.1 ± 2.9	60.4 ± 7.6	5.67	114.3	9.10	107.7
			**0.8**	66.2 ± 4.0	77.8 ± 6. 1	1.65	112.0	7.46	102.5
**PGE2 EA**	0.9928	y=1.474x+0.1612	**0.02**	59.9 ± 3.1	61.2 ± 6.0	4.72	104.9	10.3	95.9
			**0.2**	49.6 ± 5.0	60.2 ± 7.3	9.41	103.0	9.16	101.7
			**0.8**	59.4 ± 4.7	73.8 ± 4.2	1.38	100.9	4.67	102.8
**PGF2a EA**	0.9986	y=24.668x+1.0957	**0.02**	51.1 ± 3.9	47.6 ± 2.2	5.17	93.8	12.8	88.1
			**0.2**	51.1 ± 2.9	53.7 ± 7.5	5.36	111.7	7.42	104.8
			**0.8**	56.5 ±3.3	71.6 ± 5.4	1.79	110.0	6.57	101.9
**5 6 DHET**	0.9931	y=0.733x+0.0635	**0.02**	89.1 ± 9.1	43.2 ± 6.1	14.7	92.5	11.00	97.9
			**0.2**	70.4 ± 2.7	74.9 ± 11.5	5.94	97.7	10.2	99.4
			**0.8**	68.5 ± 4.0	86.8 ± 5.6	4.34	96.0	10.9	100.3
**8 9 DHET**	0.998	y=1.6982x+0.1361	**0.02**	82.5 ± 9.4	55.0 ± 4.3	9.13	95.6	12.7	88.3
			**0.2**	75.8 ± 2.9	74.0 ± 11.4	9.03	113.8	8.4	101.0
			**0.8**	70.0 ± 5.3	92.9 ± 5.4	4.41	111.9	9.01	100.7
**11 12 DHET**	0.9945	y=4.4257x+0.3991	**0.02**	92.7 ± 9.0	55.8 ± 3.7	6.60	100.0	13.6	102.6
			**0.2**	76.6 ± 2.4	80.9 ± 10.6	8.97	111.1	11.2	100.5
			**0.8**	70.2 ± 4.8	93.9 ± 6.5	3.79	110.6	9.54	98.9
**14 15 DHET**	0.9942	y=1.7111x+0.1713	**0.02**	33.2 ± 5.8	20.9 ± 2.9	8.37	90.0	14.2	86.9
			**0.2**	24.7 ± 1.8	23.3 ± 3.4	12.01	103.3	6.75	95.6
			**0.8**	25.3 ± 2.2	35.8 ± 2.5	6.38	109.4	10.5	103.7

**12 HPETE**	0.9311	y=0.2845x+0.0625	**0.02**	35.4 ± 4.4	72.2 ± 21.8	12.36	92.3	26.8	100.8
			**0.2**	23.7 ± 3.2	40.2 ± 8.1	14.7	102.8	18.8	111.4
			**0.8**	18.0 ± 1.8	70.5 ± 20.3	5.30	96.2	15.1	85.2
**5 HETE**	0.9906	y=2.5887x+0.2659	**0.02**	68.9 ± 11.2	57.3 ± 3.2	10.2	94.3	5.58	100.1
			**0.2**	84.2 ± 7.6	80.2 ± 10.3	13.4	111.3	11.0	104.8
			**0.8**	85.5 ± 7.9	99.6 ± 7.2	7.76	105.7	13.8	103.4
**8 HETE**	0.9979	y=3.1007x+0.1063	**0.02**	66.4 ± 19.3	80.0 ± 7.5	13.5	105.9	13.0	102.4
			**0.2**	86.2 ± 7.5	81.9 ± 11.2	8.38	103.3	7.86	100.9
			**0.8**	67.6 ± 4.3	98.8 ± 9.1	3.25	101.2	4.72	97.5
**9 HETE**	0.9953	y=0.897x+0.0743	**0.02**	74.3 ± 21.2	58.2 ± 8.7	13.9	102.7	10.4	97.6
			**0.2**	74.5 ± 6.9	83.5 ± 13.5	7.46	106.8	7.43	99.6
			**0.8**	65.7 ± 5.2	99.6 ± 8.2	4.16	108.2	7.34	97.8
**11 HETE**	0.9999	y=7.9138x+0.0967	**0.02**	64.3 ± 11.4	62.8 ± 3.3	6.67	102.9	6.30	104.5
			**0.2**	82.4 ± 8.7	75.9 ± 9.3	13.8	106.9	9.32	103.0
			**0.8**	73.4 ± 4.4	96.2 ± 6.0	3.33	98.1	7.57	101.3
**12 HETE**	0.9974	y=3.1096x+1.7504	**0.02**	87.1 ± 9.1	97.3 ± 7.6	14.5	105.8	10.3	105.0
			**0.2**	85.1 ± 1.8	90.6 ± 15.2	1.77	104.1	5.23	104.0
			**0.8**	84.5 ± 3.6	102.9 ± 12.7	5.19	106.9	4.50	103.7
**15 HETE**	0.9999	y=1.9007x+0.1214	**0.02**	54.1 ± 24.0	77.8 ± 2.7	1.93	113.5	12.6	101.7
			**0.2**	90.6 ± 10.5	85.4 ± 10.9	14.9	97.8	10.3	98.1
			**0.8**	76.6 ± 4.8	100.0 ± 8.5	1.70	92.8	6.47	99.2
**16 HETE**	0.9999	y=2.3239x-0.0024	**0.02**	86.2 ± 8.7	55.3 ± 4.3	9.13	96.6	13.5	90.7
			**0.2**	75.1 ± 4.8	78.0 ± 9.0	8.76	105.3	8.84	99.1
			**0.8**	73.7 ± 5.1	100.4 ± 8.2	4.33	107.5	7.91	104.2
**19 HETE**	0.9998	y=1.1225x+0.008	**0.02**	98.8 ± 13.9		6.41	110.2	15.2	101.2
			**0.2**	82.1 ± 3.7	56.0 ± 7.3	8.69	104.8	7.12	95.0
			**0.8**	76.0 ± 3.6	74.2 ± 5.5	4.22	105.3	10.3	100.6
**20 HETE**	0.9997	y=0.3259x-0.0008	**0.02**	89.6 ± 9.1	53.6 ± 11.3	11.8	101.6	20.6	101.8
			**0.2**	69.3 ± 8.8	81.4 ± 13.1	11.8	98.8	8.67	99.9

			**0.8**	78.7 ± 5.9	98.8 ± 7.4	2.92	97.7	10.3	100.4
**5 6 EET**	0.9999	y=1.1641x+0.0144	**0.02**	103.2 ± 16.1	60.5 ± 11.8	13.6	99.3	12.9	91.7
			**0.2**	99.0 ± 3.8	108.6 ± 16.0	4.78	89.2	10.6	98.7
			**0.8**	92.2 ± 6.4	116.4 ± 8.4	9.72	113.1	13.8	97.2
**8 9 EET**	0.9909	y=0.8962x+0.0876	**0.02**	58.2 ± 9.8	46.3 ± 4.1	10.5	97.8	12.6	93.4
			**0.2**	71.8 ± 7.2	78.6 ± 8.7	13.3	105.4	9.93	104.0
			**0.8**	61.8 ± 3.7	96.6 ± 7.1	4.82	107.7	12.9	104.6
**11 12 EET**	0.991	y=0.742x+0.0742	**0.02**	79.5 ± 12.5	43.8 ± 7.5	11.2	104.3	10.9	88.8
			**0.2**	80.6 ± 6.8	67.8 ± 9.5	9.53	102.8	11.9	100.7
			**0.8**	78.6 ± 8.3	94.9 ± 9.96	8.56	111.8	13.7	99.5
**14 15 EET**	0.9954	y=1.6256x+0.0427	**0.02**	72.8 ± 3.2	52.0 ± 6.2	12.1	104.2	8.24	110.9
			**0.2**	75.1 ± 10.8	76.2 ± 11.2	11.0	102.7	7.02	99.0
			**0.8**	67.3 ± 6.3	110.7 ± 8.0	8.75	111.6	9.98	101.9
**5 HPETE**	0.9996	y=0.3988x+0.0194	**0.02**	30.9 ± 5.5	37.5 ± 4.7	11.5	92.6	13.5	96.9
			**0.2**	22.5 ± 3.3	34.9 ± 2.8	14.9	102.0	14.1	94.5
			**0.8**	24.8 ± 2.9	54.7 ± 4.1	7.19	103.4	14.0	100.8
**5 6 EET EA**	0.9995	y=0.1658x+0.0047	**0.02**	60.0 ± 7.6	51.5 ± 5.4	10.2	92.9	12.6	98.2
			**0.2**	63.5 ± 5.9	68.2 ± 10.0	7.00	99.9	11.9	98.7
			**0.8**	69.5 ± 7.3	93.4 ± 7.6	5.06	93.8	12.2	101.3
**14 15 EET G**	0.9947	y=1.3893x+0.1077	**0.02**	79.9 ± 14.5	51.1 ± 7.5	13.3	102.0	12.9	98.8
			**0.2**	73.7 ± 7.6	69.9 ± 10.2	10.8	94.3	11.7	94.1
			**0.8**	65.9 ± 5.9	99.8 ± 9.0	12.2	106.8	14.5	103.1
**PEA**	0.9992	y=2.2471x+0.0495	**0.02**	75.1 ± 5.3	45.1 ± 2.5	13.6	105.1	14.8	102.8
			**0.2**	75.3 ± 10.3	38.7 ± 5.2	3.67	111.2	13.0	108.7
			**0.8**	82.2 ± 1.9	72.2 ± 2.1	12.5	104.0	9.98	97.9
**OEA**	0.9706	y=2.7102x+1.0386	**0.02**	51.9 ± 16.6	62.5 ± 4.6	2.84	101.9	12.6	101.2
			**0.2**	66.8 ± 8.5	68.2 ± 6.5	11.2	107.8	6.62	97.2
			**0.8**	52.8 ± 6.0	92.0 ± 4.6	5.56	112.0	15.6	99.4
**AEA**	0.9956	y=1.075x+0.1339	**0.02**	33.6 ± 7.8	72.2 ± 2.1	14.8	113.3	3.76	105.8

			**0.2**	55.8 ± 8.6	78.4 ± 10.2	7.89	110.8	9.33	106.0
			**0.8**	57.4 ± 4.8	111.2 ± 8.0	5.46	103.1	7.08	100.8
**2-AG**	0.9904	y=3.9296x+3.7887	**0.02**	54.1 ± 11.0	101.8 ± 6.6	11.0	94.7	15.1	100.0
			**0.2**	51.03± 6.2	133.0 ± 19.0	6.47	113.3	13.3	111.3
			**0.8**	50.9 ± 0.9	87.1 ± 8.5	12.0	104.2	10.6	110.4
**NADA**	0.9961	y=18.129x-1.1995	**0.02**	91.7 ± 7.7	45.4 ± 7.4	7.94	96.2	12.6	91.9
			**0.2**	65.4 ± 10.3	85.8 ± 15.5	8.17	99.5	10.3	103.7
			**0.8**	61.5 ± 4.3	109.5 ± 11.6	5.19	101.5	11.4	100.5

Supplementary Table S2. Levels of individual analytes measured in spinal cord, DRGs, knee joint, brain regions; frontal cortex, midbrain, hippocampus, rest of cortex, rest of brain and plasma in saline and MIA-treated rats.

							**Brain Regions**											
	**Knee Joint**		**DRGs (L3-L5)**		**Spinal Cord**		**Frontal Cortex**		**Midbrain**		**Hippocampus**		**Rest of Cortex**		**Rest of Brain**		**Plasma**	
	**Saline**	**MIA**	**Saline**	**MIA**	**Saline**	**MIA**	**Saline**	**MIA**	**Saline**	**MIA**	**Saline**	**MIA**	**Saline**	**MIA**	**Saline**	**MIA**	**Saline**	**MIA**
**PGD2/ PGE2**	39 ± 5	43 ± 3	270 ± 100	187 ± 42	30 ± 6	33 ± 9	27 ± 5	30 ± 6	19 ± 5	18 ± 2	14 ± 1	13 ± 2	32 ± 4	28 ± 1	13 ± 1	15 ± 2	2 ± 1	3 ± 0.5
**TXB2**	8 ± 1	9 ± 1	190 ± 68	218 ± 45	53 ± 5	61 ± 18	7 ± 2	9 ± 3	7 ± 1	7 ± 1	5 ± 2	3 ± 1	7 ± 1	6 ± 1	9 ± 1	11 ± 2	5 ± 2	7 ± 3
**13-HODE**	49 ± 7	61 ± 7	86 ± 29	95 ± 36	4 ± 1	2.5 ± 1.6	0.6 ± 0.1	1 ± 0.3	1 ± 0.3	0.6 ± 0.1	0.7 ± 0.1	1 ± 0.2*	1 ± 0.6	2 ± 0.5	2 ± 0.3	2 ± 0.3	6 ± 0.7	8 ± 1
**9-HODE**	19 ± 4	22 ± 4	86 ± 34	79 ± 21	4 ± 1	2.7 ± 2.6	0.5 ± 0.2	1 ± 0.3	BLOQ	0.4 ± 0.2	BLOQ	0.5 ± 0.2	1 ± 0.3	1 ± 0.1	1 ± 0.1	1 ± 0.1	3 ± 0.4	3 ± 0.5
**13-oxoODE**	0.4 ± 0.1	0.5 ± 0.1	ND	37 ± 16	ND	ND	ND	ND	ND	ND	ND	ND	ND	ND	ND	ND	0.3 ± 0.1	0.4 ± 0.1
**9-oxoODE**	0.5 ± 0.1	0.5 ± 0.1	ND	ND	ND	ND	ND	ND	ND	ND	ND	ND	ND	ND	ND	ND	0.5 ± 0.1	0.5 ± 0.1
**15-HETE**	6 ± 1	11 ± 2	5 ± 2	31 ± 12*	4 ± 2	BLOQ*	1 ± 0.2	2 ± 0.3	1 ± 0.1	0.6 ± 0.1	1 ± 0.2	1 ± 0.3	1 ± 0.1	1 ± 0.1	1 ± 0.1	1 ± 0.1	0.6 ± 0.1	1 ± 0.3
**12-HETE**	10 ± 1	23 ± 5*	262 ± 67	428 ± 144	16 ± 2	17 ± 4	3 ± 1	4 ± 1	5 ± 1	6 ± 2	3 ± 1	4 ± 1	3 ± 0.5	4 ± 1	7 ± 1	8 ± 2	8 ± 3	25 ± 8
**11-HETE**	4 ± 0.5	4 ± 0.4	40 ± 20	39 ± 11	14 ± 1	18 ± 4	2 ± 0.3	5 ± 2	1 ± 0.1	1 ± 0.1	1 ± 0.6	1 ± 0.1	2 ± 1	1 ± 0.2	1 ± 0.4	1 ± 0.5	2 ± 0.4	2 ± 0.7
**8-HETE**	1 ± 0.2	1 ± 0.3	BLOQ	BLOQ	BLOQ	BLOQ	0.3 ± 0.1	0.5 ± 0.2	0.2 ± 0.1	0.3 ± 0.1	0.2 ± 0.1	0.3 ± 0.1	0.3 ± 0.1	0.2 ± 0.1	0.2 ± 0.1	0.3 ± 0.1	0.1 ± 0.03	0.1 ± 0.03
**5-HETE**	1 ± 0.2	1 ± 0.1	10 ± 5	18 ± 6	2 ± 1	2 ± 1	1 ± 0.1	1 ± 0.2	1 ± 0.1	1 ± 0.1	0.6 ± 0.1	0.6 ± 0.1	0.5 ± 0.1	0.5 ± 0.1	0.5 ± 0.1	0.6 ± 0.1	0.3 ± 0.02	0.4 ± 0.1
**AraGly**	0.1 ± 0.02	0.1 ± 0.02	BLOQ	BLOQ	13 ± 3	18 ± 8	0.5 ± 0.1	0.5 ± 0.1	0.6 ± 0.1	0.7 ± 0.1	0.7 ± 0.3	0.7 ± 0.2	0.8 ± 0.1	0.8 ± 0.1	0.6 ± 0.1	0.7 ± 0.1	0.02 ± 0.002	0.03 ± 0.003
**AEA**	2 ± 0.4	2 ± 0.3	14 ± 6	13 ± 4	9 ± 1	21 ± 11	6 ± 1	5 ± 0.3	6 ± 1	5 ± 0.5	6 ± 1	5 ± 0.5	5 ± 0.5	4 ± 0.3*	4 ± 1	4 ± 0.5	0.7 ± 0.1	0.7 ± 0.1
**2-AG**	1 ± 0.2	1 ± 0.1	4 ± 1	3 ± 1	24 ± 3	24 ± 3	4 ± 0.3	4 ± 1	11 ± 1	10 ± 1	9 ± 1	7 ± 0.5*	5 ± 0.5	4 ± 0.3	5 ± 0.5	6 ± 0.6	31 ± 0.3	43 ± 8
**PEA**	6 ± 0.5	6 ± 1	323 ± 93	163 ± 49	176 ± 22	299 ± 65	46 ± 7	40 ± 9	47 ± 9	55 ± 5	33 ± 5	32 ± 4	16 ± 2	17 ± 2	18 ± 3	19 ± 2	3 ± 0.3	3 ± 0.4
**OEA**	4 ± 0.4	4 ± 0.5	122 ± 35	79 ± 24	128 ± 14	184 ± 39	35 ± 5	31 ± 5	41 ± 9	44 ± 3	26 ± 4	21 ± 2	15 ± 2	15 ± 2	19 ± 2	19 ± 2	5 ± 0.5	5 ± 0.3
**LA**	480 ± 61	471 ± 36	230 ± 82	303 ± 95	154 ± 14	135 ± 19	63 ± 11	49 ± 6	60 ± 15	55 ± 9	41 ± 9	39 ± 9	59 ± 3	51 ± 4	55 ± 5	58 ± 4	332 ± 23	352 ± 32
**AA**	44 ± 4	46 ± 3	376 ± 126	261 ± 74	156 ± 28	162 ± 21	328 ± 29	338 ± 18	354 ± 84	318 ± 38	355 ± 35	301 ± 31	285 ± 17	239 ± 20	212 ± 16	233 ± 22	25 ± 1	26 ± 2

Data are expressed as mean ± SEM. All values in tissue are expressed as pmol/g except for AA in nmol/g and in plasma values are expressed as nmol/L except for AA in μmol/L. ND = not detected, BLOQ = below level of quantification. Statistical comparisons between groups were performed using a Mann Whitney test where *$p<0.05$ compared to saline-treated rats
